# Supplementary material for: Recent Progress in the Discovery and Development of Monoclonal Antibodies against Viral Infections
Source: Biomedicines. 2022 Aug 2;10(8):1861. doi: 10.3390/biomedicines10081861 (PMC9405509; doi:10.3390/biomedicines10081861)
Supplement: Supplementary file 1 [file biomedicines-10-01861-s001.zip › biomedicines-1764215-supplementary.pdf]

Table S1. Baltimore classification of viruses based on rare to pandemic diseases caused by viruses.

G	type	Genus, Family	host	Transmission	Disease	Virus
I	dsDNA viruses					
		Lymphocryptovirus, Herpesviridae	Human, monkeys	Zoonosis, animal bite	Encephalitis	Cercopithecine herpesvirus
		Lymphocryptovirus, Herpesviridae	Human	Contact, saliva	Mononucleosis	Epstein-Barr virus
		Orthohepadnavirus, Hepadnaviridae	Human, Chimpanzees	Sexual contact, blood	Hepatitis	Hepatitis B virus
		Cytomegalovirus, Herpesviridae	Human	Contact, urine, saliva	Mononucleosis, pneumonia	Human cytomegalovirus
		Simplexvirus, Herpesviridae	Human	Sexual contact, saliva	Skin lesions	Human herpesvirus 1
		Simplexvirus, Herpesviridae	Human	Sexual contact, saliva	Skin lesions	Human herpesvirus 2
		Roseolovirus, Herpesviridae	Human	Respiratory, contact	Skin lesions	Human herpesvirus 6
		Roseolovirus, Herpesviridae	Human	Respiratory, contact	Skin lesions	Human herpesvirus 7
		Rhadinovirus, Herpesviridae	Human	Sexual contact, saliva	Skin lymphoma	Human herpesvirus 8
		Mupapillomavirus, Papillomaviridae	Human	Contact	Skin warts	Human papillomavirus 1
		Alphapapillomavirus, Papillomaviridae	Human	Contact	Skin warts	Human papillomavirus 2
		Alphapapillomavirus, Papillomaviridae	Human	Sexual	Genital warts, cervical cancer	Human papillomavirus 16,18
		Polyomavirus, Polyomaviridae	Human	Fecal-oral or urine	Encephalitis	JC polyomavirus
		Polyomavirus, Polyomaviridae	Human	Fecal-oral or urine	Encephalitis	KI Polyomavirus
		Polyomavirus, Polyomaviridae	Human	-	Merkel cell carcinoma	Merkel cell polyomavirus
		Molluscipoxvirus, Poxviridae	Human	Contact	Skin lesions	Molluscum contagiosum virus
		Orthopoxvirus, Poxviridae	Human, mouse, prairie dog	Zoonosis, contact	Skin lesions	Monkeypox virus
		Parapoxvirus, Poxviridae	Human, mammals	Zoonosis, contact	Skin lesions	Orf virus
		Varicellovirus, Herpesviridae	Human	Respiratory, contact	Varicella	Varicella-zoster virus
		Orthopoxvirus, Poxviridae	Human	Respiratory	Variola	Variola virus
II	ssDNA viruses					

		Erythrovirus, Parvoviridae	Human	Respiratory	Skin lesion	Human parvovirus B19
III	dsRNA viruses					
		Seadornavirus, Reoviridae	Human, cattle, pig, mosquitoes	Zoonosis, arthropod bite	Encephalitis	Banna virus
		Rotavirus, Reoviridae	Human	Fecal-oral	Gastroenteritis	Rotavirus A
		Rotavirus, Reoviridae	Human	Fecal-oral	Gastroenteritis	Rotavirus B
		Rotavirus, Reoviridae	Human	Fecal-oral	Gastroenteritis	Rotavirus C
IV	(+)ssRNA viruses					
		Kobuvirus, Picornaviridae	Human	Fecal-oral	Gastroenteritis	Aichi virus
		Alphavirus, Togaviridae	Human, marsupials, mosquitoes	Zoonosis, arthropod bite	Fever, joint pain	Barmah forest virus
		Alphavirus, Togaviridae	Human, monkeys, mosquitoes	Zoonosis, arthropod bite	Fever, joint pain	Chikungunya virus
		Enterovirus, Picornaviridae	Human	Fecal-oral	Meningitis, myocarditis, paralysis	Coxsackievirus
		Flavivirus, Flaviviridae	Human, mosquitoes	Zoonosis, arthropod bite	Hemorrhagic fever	Dengue virus
		Alphavirus, Togaviridae	Human, birds, mosquitoes	Zoonosis, arthropod bite	Encephalitis	Eastern equine encephalitis virus
		Enterovirus, Picornaviridae	Human	Fecal-oral	Common cold	Echovirus
		Cardiovirus, Picornaviridae	Human, mouse, rat, pig	Zoonosis	Encephalitis	Encephalomyocarditis virus
		Hepatovirus, picornaviridae	Human	Fecal-oral	Hepatitis	Hepatitis A virus
		Hepacivirus, Flaviviridae	Human	Sexual, blood	Hepatitis	Hepatitis C virus
		Hepevirus, Unassigned	Human, pig, monkeys, some rodents, chicken	Zoonosis, food	Hepatitis	Hepatitis E virus
		Mamastrovirus, Astroviridae	Human	Fecal-oral	Gastroenteritis	Human astrovirus
		Alphacoronavirus, Coronaviridae	Human	Respiratory	Respiratory	Human coronavirus
		Enterovirus, Picornaviridae	Human	Fecal-oral	Diarrhea, neurological disorder	Human enterovirus 68, 70
		Enterovirus, Picornaviridae	Human	Respiratory	Respiratory	Human rhinovirus
		Betacoronavirus, Coronaviridae	Human, bats, palm civet	Zoonosis	Respiratory	Human SARS coronavirus

		Deltaretrovirus, Retroviridae	Human	Sexual contact, maternal-neonatal	Leukemia	Human T-lymphotropic virus
		Torovirus, Coronaviridae	Human	Fecal-oral	Gastroenteritis	Human torovirus
		Flavivirus, Flaviviridae	Human, horses, birds, mosquitoes	Zoonosis, arthropod borne	Encephalitis	Japanese encephalitis virus
		Flavivirus, Flaviviridae	Human, horses, birds, mosquitoes	Zoonosis, arthropod borne	Encephalitis	Kunjin virus
		Flavivirus, Flaviviridae	Human, ticks	Zoonosis, arthropod borne	Encephalitis	Langat virus
		Norovirus, Caliciviridae	Human	Fecal-oral	Gastroenteritis	Lordsdale virus
		Flavivirus, Flaviviridae	Human, mammals, ticks	Zoonosis, arthropod bite	Encephalitis	Louping ill virus
		Alphavirus, Togaviridae	Human, mosquitoes	Zoonosis, arthropod bite	Fever, joint pain	Mayaro virus
		Betacoronavirus, Coronaviridae	Human, Tomb bat	Zoonosis	Respiratory	MERS coronavirus
		Morbilivirus, Paramyxoviridae	Human	Respiratory	Fever, rash	Measles virus
		Cardiovirus, Picornaviridae	Human, mouse, rabbit	Zoonosis	Encephalitis	Mengo encephalomyocarditis virus
		Flavivirus, Flaviviridae	Human, mosquitoes	Zoonosis, arthropod bite	Encephalitis	Murray valley encephalitis virus
		Norovirus, Caliciviridae	Human	Fecal-oral	Gastroenteritis	Norwalk virus
		Alphavirus, Togaviridae	Human, mosquitoes	Zoonosis, arthropod bite	Fever, joint pain	O'nyong-nyong virus
		Enterovirus, Picornaviridae	Human, mammals	Fecal-oral	Poliomyelitis	Poliovirus
		Alphavirus, Togaviridae	Human, mosquitoes, marsupials	Zoonosis, arthropod bite	Fever, joint pain	Ross river virus
		Rubivirus, Togaviridae	Human	Respiratory	Rubella	Rubella virus
		Alphavirus, Togaviridae	Human, horse, pig, mosquitoes	Zoonosis, arthropod bite	Fever, joint pain	Sagiyama virus
		Salivirus, Picornaviridae	Human		Gastroenteritis	Salivirus A
V	(-)ssRNA viruses					
		Lyssavirus, Rhabdoviridae	Human, bats	Zoonosis, animal bite	Fatal encephalitis	Australian bat lyssavirus
		Orthobunyavirus, Bunyaviridae	Human, mosquitoes	Zoonosis, arthropod bite	Encephalitis	Bunyamwera virus
		Orthobunyavirus, Bunyaviridae	Human, deer, mosquitoes, tamias	Zoonosis, arthropod bite	Encephalitis	Bunyavirus La Crosse

	Orthobunyavirus, Bunyaviridae	Human, rodents, mosquitoes	Zoonosis, arthropod bite	Encephalitis	Bunyavirus snowshoe hare
	Vesiculovirus, Rhabdoviridae	Human, sandflies	Zoonosis, athropod bite	Encephalitis	Chandipura virus
	Nairovirus, Bunyaviridae	Human, vertebrates, ticks	Zoonosis, arthropod bite	Hemorrhagic fever	Crimean-Congo hemorrhagic fever virus
	Thogotovirus, Orthomyxoviridae	Human, ticks	Zoonosis, arthropod bite	Fever, encephalitis	Dhori virus
	Nairovirus, Bunyaviridae	Human, ticks	Zoonosis, arthropod bite	Thrombocytopaenia	Dugbe virus
	Lyssavirus, Rhabdoviridae	Human, mammals	Zoonosis, animal bite	Fatal encephalitis	Duvenhage virus
	Ebolavirus, Filoviridae	Human, monkeys, bats	Zoonosis, contact	Hemorrhagic fever	Ebolavirus
	Hantavirus, Bunyaviridae	Human, rodents	Zoonosis, urine, saliva	Renal or respiratory syndrome	Hantaan virus
	Henipavirus, paramyxoviridae	Human, horse, bats	Zoonosis, animal bite	Encephalitis	Hendra virus
	Deltavirus, Unassigned	Human	Sexual contact, blood	Hepatitis	Hepatitis delta virus
	Respirovirus, Paramyxoviridae	Human	Respiratory	Respiratory	Human parainfluenza
	Orthopneumovirus, Pneumoviridae	Human	Respiratory	Respiratory	Human respiratory syncytial virus
	Influenzavirus A, Orthomyxoviridae	Human, birds, pigs	Respiratory or Zoonosis, animal contact	Flu	Influenza A virus
	Influenzavirus B, Orthomyxoviridae	Human	Respiratory	Flu	Influenza B virus
	Influenzavirus C, Orthomyxoviridae	Human	Respiratory	Flu	Influenza C virus
	Vesiculovirus, Rhabdoviridae	Human, sandflies, gerbils	Zoonosis, arthropod bite	Undocumented, encephalitis?	Isfahan virus
	Arenavirus, Arenaviridae	Human, rodents	Zoonosis, fomite	Hemorrhagic fever	Junin arenavirus
	Lyssavirus, Rhabdoviridae	Human, mammals	Zoonosis, animal bite	Fatal encephalitis	Lagos bat virus
	Marburgvirus, Filoviridae	Human, monkeys, bats	Zoonosis, fomite	Hemorrhagic fever	Lake Victoria marburgvirus
	Arenavirus, Arenaviridae	Human, rats	Zoonosis, fomites	Hemorrhagic fever	Lassa virus
	Arenavirus, Arenaviridae	Human, rodents	Zoonosis, fomite	Encephalitis	Lymphocytic choriomeningitis virus
	Arenavirus, Arenaviridae	Human, monkeys, mouse	Zoonosis, fomite	Encephalitis	Machupo virus
	Lyssavirus, Rhabdoviridae	Human, rodents, cat, dog shrew	Zoonosis, animal bite	Encephalitis	Mokola virus
	Rubulavirus, Paramyxoviridae	Human	Respiratory, saliva	Mumps	Mumps virus

		Hantavirus, Bunyavirus	Human, mouse	Zoonosis, urine, saliva	Hemorrhagic fever	New York virus
		Henipavirus, Paramyxoviridae	Human, bats	Zoonosis, animal bite	Encephalitis	Nipah virus
		Orthobunyavirus, Bunyaviridae	Human, wild animals(sloths)	Zoonosis, arthropod bite	Fever, joint pain	Oropouche virus
		Arenavirus, Arenaviridae	Human, rat, guinea pig	Zoonosis, fomite	Hemorrhagic fever	Pichinde virus
		Phlebovirus, Bunyaviridae	Human, sandflies	Zoonosis, arthropod bite	Hemorrhagic fever	Punta toro phlebovirus
		Hantavirus, Bunyavirus	Human, bank vole	Zoonosis, urine, saliva	Hemorrhagic fever	Puumala virus
		Lyssavirus, Rhabdoviridae	Human, mammals	Zoonosis, animal bite	Fatal encephalitis	Rabies virus
		Phlebovirus, Bunyaviridae	Human, mammals, mosquitoes, sandflies	Zoonosis, arthropod bite	Hemorrhagic fever	Rift valley fever virus
		Phlebovirus, Bunyaviridae	Human, sandflies	Zoonosis, arthropod bite	Hemorrhagic fever	Sandfly fever sicilian virus
		Hantavirus, Bunyavirus	Human, rats	Zoonosis, urine, saliva	Hemorrhagic fever	Seoul virus
		Phlebovirus, Bunyaviridae	Human, mosquitoes	Zoonosis, arthropod bite	Hemorrhagic fever	Toscana virus
		Phlebovirus, Bunyaviridae	Human, ticks	Zoonosis, arthropod bite	Hemorrhagic fever	Uukuniemi virus
		Vesiculovirus, Rhabdoviridae	Human, cattle, horse, pig, flies	Zoonosis, athropod bite	Encephalitis	Vesicular stomatitis virus
VI	ssRNA-RT viruses					
		Lentivirus, Retroviridae	Human	Sexual contact, blood	AIDS	Human immunodeficiency virus
VII	dsDNA-RT viruses					

Table S2. Mabs related to specific antigen in viral infections followed by clinical phase.

Row	Status	Study Title	Conditions	Interventions and target antigen	Phase
	**Zika virus** a member of *Flaviviridaeis* (+ssRNA viruses-Grroup IV)				
1	Completed	Safety and Tolerability of an Antibody Against Zika Virus (Tyzivumab) in Humans	Treatment of Acute Zika Virus Infection	• Drug: Tyzivumab<br>ZIKV monoclonal antibody (mAb) surface-exposed envelope (E) protein of the virus	Phase 1
2	Recruiting	Safety and Tolerability of an Antibody Against Zika Virus (Tyzivumab) in ZIKV Infected Patients	Treatment of Acute Zika Virus Infection	• Biological: Tyzivumab<br>• Other: Placebo	Phase 1
	**The human immunodeficiency viruses (HIV)** are a subgroup of Retroviridae				
1	Completed	The Safety and Effectiveness of Human Monoclonal Antibody, F105, in the Treatment of HIV	HIV Infections	• Drug: F105 Monoclonal Antibody (Human) an anti-gp120	Phase 1
2	Completed	Safety and Virologic Effect of a Human Monoclonal Antibody (VRC01) Administered Intravenously to Adults During Early Acute HIV Infection	HIV Infections	• Biological: VRC01 (targeting specific B cell receptors)<br>• Biological: Placebo for VRC01<br>• Drug: Antiretroviral therapy (ART) (regimen will vary within countries and by patient)	Phase 1
3	Completed<br>Has Results	Evaluating the Safety, Tolerability, and Effect of a Human Monoclonal Antibody (VRC01) on Markers of HIV Persistence in HIV-Infected Adults Receiving Antiretroviral Therapy (ART)	HIV Infections	• Biological: VRC01 (targeting specific B cell receptors)<br>• Biological: Placebo	Phase 1
4	Active, not recruiting	Evaluating the Safety, Tolerability, Pharmacokinetics, and Antiviral Activity of the Monoclonal Antibody PGT121.414.LS Administered Alone and in Combination With VRC07-523LS Via Intravenous or Subcutaneous Infusions in Healthy, HIV-uninfected Adult Participants	HIV Infections	• Biological: PGT121.414.LS targeting V3-glycan<br>• Biological: VRC07-523LS**(targeting specific B cell receptors CD4 binding site)**	Phase 1
5	Completed	Safety, PK and Antiviral Activity of PGT121 Monoclonal Antibody in HIV-uninfected and HIV-infected Adults	HIV Infection	• Biological: PGT121 3mg/kg IV<br>• Biological: PGT121 10mg/kg IV<br>• Biological: PGT121 30mg/kg IV (targeting V3-glycan)<br>• (and 2 more...)	Phase 1
6	Completed	Evaluating the Safety and Serum Concentrations of a Human Monoclonal Antibody, VRC-HIVMAB075-00-AB	HIV Infections	• Biological: VRC07-523LS(**targeting specific B cell receptors CD4 binding site)**	Phase 1

		(VRC07-523LS), Administered in Multiple Doses and Routes to Healthy, HIV-uninfected Adults		• Biological: Placebo	
7	Completed	Evaluating the Safety, Tolerability, Pharmacokinetics, and Antiviral Activity of Combinations of Monoclonal Antibodies PGT121, PGDM1400, 10-1074, and VRC07-523LS Administered Via Intravenous Infusion in Healthy, HIV-uninfected Adult Participants	HIV Infections	• Biological: PGT121(targeting V3-glycan)<br>• Biological: PGDM1400 (targeting V1-V2 glycan)<br>• Biological: 10-1074(targeting V3-glycan)<br>• Biological: VRC07-523LS(**targeting specific B cell receptors CD4 binding site)**	Phase 1
8	Completed<br>Has Results	Evaluating the Safety and Pharmacokinetics of VRC01, VRC01LS, and VRC07-523LS, Potent Anti-HIV Neutralizing Monoclonal Antibodies, in HIV-1-Exposed Infants	HIV Infections	• Biological: VRC01<br>• Biological: VRC01LS<br>• Biological: VRC07-523LS<br><br>(**targeting specific B cell receptors CD4 binding site)**	Phase 1
9	Completed	Phase I/II, Open-Label Trial of Three Monoclonal Antibodies	HIV Infections	• Drug: Potent HAART during acute or early HIV-1 infection<br><br>combination of two nucleoside reverse transcriptase inhibitors (typically tenofovir-emtricitabine) plus one non-nucleoside reverse transcriptase inhibitor or integrase strand transfer inhibitor.	Phase 1<br>Phase 2
10	Completed	A Study of Anti-HIV Monoclonal Antibody KD-247	HIV Infections	• Drug: KD-247 (targeting V3-glycan)<br>• Drug: Physiological saline	Phase 1
11	Completed<br>Has Results	Safety and Therapeutic Efficacy of the VRC01 Antibody in Patients Who Initiated Antiretroviral Therapy During Early Acute HIV Infection	HIV Infections	• Biological: VRC01 (**targeting specific B cell receptors CD4 binding site)**<br>• Biological: Placebo for VRC01	Phase 2
12	Completed	A Clinical Trial of PGDM1400 and PGT121 and VRC07-523LS Monoclonal Antibodies in HIV-infected and HIV-uninfected Adults	HIV Infections	• Biological: PGDM1400/Placebo (3mg/kg IV)<br>• Biological: PGDM1400/Placebo (10mg/kg IV)<br>• Biological: PGDM1400/Placebo (30mg/kg IV)<br>• (and 5 more...)<br><br>(targeting V1-V2 glycan)	Phase 1
13	Completed	Evaluating the Safety and Antiviral Activity of Monoclonal Antibody VRC01 in HIV-Infected Infants Receiving Combination Antiretroviral Therapy	HIV Infections	• Biological: VRC01 (**targeting specific B cell receptors CD4 binding site)**<br>• Drug: Combination Antiretroviral Therapy (cART)	Phase 1<br>Phase 2
14	Completed	Evaluating the Safety and Efficacy of the VRC01 Antibody	HIV Infections	• Biological: VRC01	Phase 2

		in Reducing Acquisition of HIV-1 Infection in Women		• Biological: Placebo for VRC01<br><br>**(targeting specific B cell receptors CD4 binding site)**	
15	Completed	Evaluating the Safety and Efficacy of the VRC01 Antibody in Reducing Acquisition of HIV-1 Infection Among Men and Transgender Persons Who Have Sex With Men	HIV Infections	• Biological: VRC01<br>• Biological: Placebo for VRC01<br><br>**(targeting specific B cell receptors CD4 binding site)**	Phase 2
16	Completed<br>Has Results	Evaluating the Safety, Pharmacokinetics, and Antiviral Activity of a Human Monoclonal Antibody (VRC01) in HIV-Infected Adults Undergoing a Brief Treatment Interruption	HIV Infections	• Biological: VRC01<br><br>(targeting specific B cell receptors CD4 binding site)	Phase 1
17	Completed	A Phase I/II Trial to Assess the Safety and Tolerance of Escalating Doses of a Human Anti-Cytomegalovirus Monoclonal Antibody (SDZ MSL-109) in Patients With the Acquired Immunodeficiency Syndrome and CMV Retinitis	Cytomegalovirus Retinitis<br><br>HIV Infections	• Drug: Sevirumab<br><br>Anti-Cytomegalovirus	Phase 1
18	Recruiting	A Phase 1/2a Study of PGT121, VRC07-523LS and PGDM1400 Monoclonal Antibodies in HIV-uninfected and HIV-infected Adults	HIV/AIDS	Biological: PGT121 + VRC07-523LS (targeting V3glycan+targeting specific B cell receptors CD4 binding site)<br><br>• Biological: PGT121 + VRC07-523LS + PGDM1400 (targeting V3glycan+targeting specific B cell receptors CD4 binding site+targeting V1-2glycan)	Phase 1<br>Phase 2
19	Completed	Safety and Pharmacokinetics of a Human Monoclonal Antibody, VRC-HIVMAB080-00-AB (VRC01LS), With Broad HIV-1 Neutralizing Activity, Administered Intravenously or Subcutaneously to Healthy Adults	Prevention of HIV Infection<br><br>HIV Antibodies<br><br>Monoclonal Antibody, Human<br><br>Neutralizing Antibody	• Biological: VRC-HIVMAB080-00-AB (VRC01LS)<br>• Biological: VRC-HIVMAB060-00-AB (VRC01)<br><br>(targeting specific B cell receptors CD4 binding site)	Phase 1
20	Completed	A Randomized, Phase I/II Trial to Assess the Safety and Antiviral Effects of Escalating Doses of A Human Anti-Cytomegalovirus Monoclonal Antibody (SDZ MSL-109) in Patients With the Acquired Immunodeficiency Syndrome and CMV Viremia and/or Viruria	Cytomegalovirus Infections<br><br>HIV Infections	• Drug: Sevirumab Anti-Cytomegalovirus Monoclonal Antibody (SDZ MSL-109)	Phase 1
21	Unknown †	Study of Single IV Administration of P2G12	HIV Infection	• Drug: P2G12 Dose Cohort 1<br>• Drug: P2G12 Dose Cohort 2	Phase 1

				• Drug: P2G12 Dose Cohort 3<br><br>Targeting gp41- MPER	
22	Recruiting	PET Imaging of Radiolabeled Anti-HIV-1 Envelope Monoclonal Antibody (VRC01)	HIV-1-infection	• Drug: [89]Zr-DFO-VRC-HIVMAB060-00-AB<br><br>(targeting specific B cell receptors CD4 binding site)	Phase 1
23	Completed	Vaginal Antibody Safety Trial: Safety Study of Monoclonal Antibodies to Reduce the Vaginal Transmission of Herpes Simplex Virus (HSV) and Human Immunodeficiency Virus (HIV)	HIV<br><br>Herpes Simplex Infections	• Drug: MB66<br><br>a multipurpose prevention technology (MPT) product with monoclonal antibodies (mAbs) against HIV-1 (VRC01-N) and HSV-1 and 2 (HSV8-N)<br><br>• Drug: Placebo Film	Phase 1
24	Completed	Evaluating the Safety and Drug Levels of an Antibody Against HIV in Healthy, HIV-Uninfected Adults	HIV Infections	• Biological: VRC01<br>• Biological: SC placebo for VRC01<br>• Biological: IV placebo for VRC01<br><br>(targeting specific B cell receptors CD4 binding site)	Phase 1
25	Completed	Study of the Safety and Pharmacokinetics of a Human Monoclonal Antibody, VRC-HIVMAB060-00-AB (VRC01), Administered Intravenously or Subcutaneously to Healthy Adults	HIV Infection<br><br>Monoclonal Antibody, Human<br><br>HIV Antibodies<br><br>(and 2 more...)	• Drug: VRC-HIVMAB060-00-AB<br><br>(targeting specific B cell receptors CD4 binding site)	Phase 1
26	Completed<br>Has Results	Studies of the Ocular Complications of AIDS (SOCA)--Monoclonal Antibody CMV Retinitis Trial (MACRT)	HIV Infections<br><br>Cytomegalovirus Retinitis	• Drug: MSL-109 Anti CMV<br>• Other: Placebo	Phase 2<br>Phase 3
27	Active, not recruiting	3BNC117-LS and 10-1074-LS in Viremic HIV-infected Individuals	Human Immunodeficiency Virus	• Drug: 3BNC117-LS (targeting specific B cell receptors CD4 binding site)<br>• Drug: 10-1074-LS (targeting V3glycan)	Phase 1
28	Completed	Safety and Tolerability of PRO 140 in HIV Uninfected Male Volunteers	HIV Infections	• Drug: PRO 140 anti-CCR5 monoclonal antibody	Phase 1
29	Completed	A Phase II/III Trial of Human Anti-CMV Monoclonal Antibody MSL 109 (MACRT)	Cytomegalovirus Retinitis<br><br>HIV Infections	• Drug: Sevirumab Anti-CMV Monoclonal	Phase 2
30	Completed	VRC 601: A Phase I, Open-Label, Dose-Escalation Study of the Safety and Pharmacokinetics of a Human Monoclonal Antibody, VRC HIVMAB060-00-AB (VRC01), With Broad HIV-1 Neutralizing	HIV-1 Infection<br><br>Neutralizing Antibody<br><br>Monoclonal Antibody	• Biological: VRC-HIVMAB060-00-AB (targeting specific B cell receptors CD4 binding site)	Phase 1

		Activity, Administered Intravenously or Subcutaneously to HIV-Infected...	(and 2 more...)		
31	Completed	3BNC117 and 10-1074 in HIV Uninfected Adults	Human Immunodeficiency Virus	• Drug: 3BNC117 (targeting specific B cell receptors CD4 binding site)<br>• Drug: 10-1074 (targeting V3glycan)<br>• Drug: Placebo	Phase 1
32	Completed<br>Has Results	Dose-Response Study of Ibalizumab (Monoclonal Antibody) Plus Optimized Background Regimen in Patients With HIV-1	HIV	• Drug: Ibalizumab (targeting CD4 receptors)	Phase 2
33	Recruiting	A Study to Evaluate the Antiviral Effect, Safety and Tolerability of GSK3810109A in Viremic Human Immunodeficiency Virus (HIV)-1 Infected Adults	HIV Infections	• Biological: GSK3810109A<br><br>an HIV-1 Specific Broadly Neutralizing Human Monoclonal Antibody	Phase 2
34	Completed<br>Has Results	Vedolizumab (Anti-alpha4beta7) in Subjects With HIV Infection Undergoing Analytical Treatment Interruption	HIV	• Biological: Entyvio (Vedolizumab) targeting alpha4beta7	Phase 1
35	Unknown †	TNX-355 With Optimized Background Therapy (OBT) in Treatment-Experienced Subjects With HIV-1	HIV Infections	• Drug: TNX-355 (anti CD-4)	Phase 2
36	Completed<br>Has Results	Phase 1, Open-label, Single Dose Study to Examine Safety, Tolerability, Pharmacokinetics and Virologic Impact of VRC01LS or VRC07-523LS in HIV-infected Viremic Adults	HIV-1	• Biological: VRC-HIVMAB080-00-AB<br>• Biological: VRC-HIVMAB075-00-AB<br><br>(targeting specific B cell receptors CD4 binding site)	Phase 1
37	Not yet recruiting	VRC 611: Human Monoclonal Antibody (mAb) VRC-HIVMAB0102-00-AB (CAP256V2LS)Administered Via Subcutaneous and Intravenous Injection in Healthy Adults	HIV	• Biological: VRC-HIVMAB0102-00-AB<br><br>(targeting specific B cell receptors CD4 binding site)	Phase 1
38	Completed	A Study of the Safety, Pharmacokinetics and Antiretroviral Activity of 10-1074	Healthy<br><br>HIV	• Biological: 3 mg/kg, single dose IV administration of 10-1074<br>• Biological: 10 mg/kg, single dose IV administration of 10-1074<br>• Biological: 30 mg/kg, single dose IV administration of 10-1074<br><br>(targeting V3glycan)	Phase 1

39	Completed	DCVax Plus Poly ICLC in Healthy Volunteers	HIV-1 Infection<br><br>HIV Infection<br><br>Healthy Volunteers	• Drug: DCVax-001 anti CD205<br>• Drug: Poly-ICLC<br>• Drug: Placebo	Phase 1
40	Completed<br>Has Results	Study of PRO 140 by Subcutaneous Administration in Adult Subjects With HIV -1 Infection	HIV -1 Infection<br><br>HIV Infections	• Drug: PRO 140 (humanized monoclonal antibody to CCR5)<br>• Drug: Placebo Comparator	Phase 2
41	Completed<br>Has Results	3BNC117 Monoclonal Antibody in HIV-infected Subjects During Treatment Interruption	HIV-1 Infection	• Drug: 3BNC117(targeting specific B cell receptors CD4 binding site)<br>• Other: ART Interruption	Phase 2
42	Completed<br>Has Results	Study of Simtuzumab in HIV and/or Hepatitis C- Infected Adults With Liver Fibrosis	Liver Fibrosis<br><br>Hepatitis C<br><br>HIV<br><br>HIV/HCV Co-infection	• Biological: Simtuzumab targeting lysyl oxidase-like 2 (LOXL2)	Phase 2
43	Recruiting	VRC 609 Study: A Phase I, Open-Label, Dose-Escalation Study of the Safety and Pharmacokinetics of a Human Monoclonal Antibody, VRC-HIVMAB091-00-AB (N6LS), Administered Intravenously or Subcutaneously With or Without Recombinant Human Hyaluronidase PH...	HIV Antibodies	• Biological: VRC-HIVMAB091-00-AB (targeting specific B cell receptors CD4 binding site)<br><br>• Biological: EDP	Phase 1
44	Completed<br>Has Results	VRC 605: Safety and Pharmacokinetics of a Human Monoclonal Antibody, VRC-HIVMAB075-00-AB (VRC07-523LS), Administered Intravenously or Subcutaneously to Healthy Adults	HIV Prevention	• Biological: VRC-HIVMAB075-00-AB (targeting specific B cell receptors CD4 binding site)	Phase 1
45	Completed	A Randomized, Double-blind, Placebo-controlled Trial, Followed by Single-arm Treatment of PRO 140 in Combination w/ Optimized Background Therapy in Treatment-Experienced HIV Subjects	HIV	• Drug: PRO 140 (humanized monoclonal antibody to CCR5)<br>• Drug: Placebo<br>• Drug: Optimized Background Regimen	Phase 2<br>Phase 3
46	Completed	3BNC117-LS First-in-Human Phase 1 Study	Human Immunodeficiency Virus	• Drug: 3BNC117-LS (targeting specific B cell receptors CD4 binding site)<br>• Drug: Placebo	Phase 1
47	Completed	Bavituximab Repeat-Dose Trial in Patients Co-Infected With Chronic Hepatitis C Virus and	Hepatitis C Virus<br><br>Hiv Infections	• Drug: bavituximab targeting phosphatidylserine (PS)	Phase 1

		Human Immunodeficiency Virus			
48	Withdrawn	PRO 140 for Human Immunodeficiency Virus Infection	HIV	• Biological: PRO 140 (humanized monoclonal antibody to CCR5)	Phase 2
49	Recruiting	10E8.4/iMab Bispecific Antibody in HIV-uninfected and HIV-infected Adults	HIV-1-infection	• Biological: 10E8.4/iMab targeting gp41 MPER	Phase 1
50	Active, not recruiting	3BNC117 and 10-1074 in ART-treated Individuals	Human Immunodeficiency Virus	• Drug: 3BNC117 LS (targeting specific B cell receptors CD4 binding site)<br>• Drug: 10-1074 targeting V3 glycan<br>• Other: Analytical treatment interruption	Phase 1
51	Completed	Investigator-Sponsored Protocol - Continued Use of Ibalizumab	HIV Infections	• Drug: ibalizumab 800mg Q2Weeks<br>• Drug: ibalizumab 2000mg Q4Weeks<br><br>Targeting CD4 receptors	Phase 2
52	Completed	Evaluating the Safety and Pharmacokinetics of VRC-HIVMAB075-00-AB (VRC07-523LS) in the Sera and Mucosae of Healthy, HIV-Uninfected Adult Participants	HIV Infections	• Biological: VRC07-523LS (targeting specific B cell receptors CD4 binding site)	Phase 1
53	Completed<br>Has Results	Ibalizumab Plus Optimized Background Regimen in Treatment-Experienced Patients With Multi-Drug Resistant HIV-1	HIV	• Drug: ibalizumab Targeting CD4 receptors<br>• Drug: Optimized Background Regimen	Phase 3
54	Completed	A Phase I Study to Evaluate the Safety and Immunogenicity of Heterologous Boost Immunizations With MVA-CMDR	HIV	• Biological: MVA-CMDR **expressing *env* and *gag-pol* genes**	Phase 1
55	Recruiting	Long-Acting Cabotegravir Plus VRC-HIVMAB075-00-AB (VRC07-523LS) for Viral Suppression in Adults Living With HIV-1	HIV Infections	• Drug: Oral Cabotegravir (CAB)<br>• Drug: Nucleoside Reverse Transcriptase Inhibitors (NRTIs)<br>• Drug: Long-Acting Injectable Cabotegravir (CAB LA)<br>• (and 2 more...)<br><br>(targeting specific B cell receptors CD4 binding site)	Phase 2
56	Not yet recruiting	Study for Evaluation of the Safety, Pharmacokinetics, and Antiviral Activity of UB-421 Subcutaneous Formulation in HIV Infected Adults	HIV-1-infection	• Biological: UB-421 SC targeting CD4 receptor on T-cells	Phase 1

57	Completed	Romidepsin Plus 3BNC117 Phase 2a Study	Human Immunodeficiency Virus (HIV)	• Drug: 3BNC117 (targeting specific B cell receptors CD4 binding site)<br>• Drug: Romidepsin	Phase 2
58	Completed<br>Has Results	VRC-HIVMAB060-00-AB (VRC01) in People With Chronic HIV Infection Undergoing Analytical Treatment Interruption	HIV	• Biological: VRC-HIVMAB060-00-AB (VRC01) (targeting specific B cell receptors CD4 binding site)	Phase 1
59	Completed	3BNC117 and 10-1074 in HIV-infected Individuals	Human Immunodeficiency Virus (HIV)	• Drug: 3BNC117 (targeting specific B cell receptors CD4 binding site)<br>• Drug: 10-1074 targeting V3 glycan<br>• Other: Analytical treatment interruption<br>• Drug: Placebo	Phase 1
60	Completed	First-in-human Study of 10-1074-LS Alone and in Combination With 3BNC117-LS	Human Immunodeficiency Virus	• Drug: 10-1074-LS targeting V3 glycan<br>• Drug: 3BNC117-LS (targeting specific B cell receptors CD4 binding site)	Phase 1
61	Completed	Combination Chemotherapy With or Without Monoclonal Antibody Therapy in Treating Patients With Previously Untreated HIV-Associated Non-Hodgkin's Lymphoma	Lymphoma	• Biological: filgrastim<br>• Biological: rituximab targeting CD20<br>• Drug: CHOP regimen<br>• (and 4 more...)	Phase 3
62	Completed	A Phase 1, Open Label, Dose-escalation Study of the Safety, Pharmacokinetics and Antiretroviral Activity of 3BNC117	Healthy<br><br>HIV	• Biological: 1 mg/kg, single dose IV administration of 3BNC117<br>• Biological: 3 mg/kg, single dose IV administration of 3BNC117<br>• Biological: 10 mg/kg, single dose IV administration of 3BNC117<br>• (and 3 more...)<br><br>(targeting specific B cell receptors CD4 binding site)	Phase 1
63	Withdrawn	Safety And Efficacy Study Of CP-675,206 In HIV-Infected Patients	Human Immunodeficiency Virus	• Drug: CP-675,206 anti-cytotoxic T lymphocyte-associated antigen 4	Phase 2
64	Completed	High Dose Therapy and Peripheral Blood Stem Cell Transplantation in HIV Related Non Hodgkin Lymphoma (NHL) at High Risk	HIV-related Lymphoma<br><br>HIV Infections	• Other: Rituximab targeting CD20 and CHOP regimen + PBSCT	Phase 2
65	Completed	Combination Therapy With 3BNC117 and 10-1074 in HIV-Infected Individuals	HIV	• Biological: 3BNC117 (targeting specific B cell receptors CD4 binding site) and 10-1074 (targeting V3 glycan)	Phase 1

				• Biological: Normal saline placebo	
66	Recruiting	Role of PLA2G1B During HIV Infection	HIV Infections	• Other: biological sample	Not Applicable
67	Completed	A Safety Study Of A Single Vaginal Administration Of P2G12 Antibody In Healthy Female Subjects	Human Immunodeficiency Virus	• Drug: P2G12 targeting gp41 MPER	Phase 1
68	Completed<br>Has Results	A Research Study to See if a Change in Therapy for HIV Infection Can Improve the Immune Response to Treatment	HIV Infections	• Drug: Kaletra + Current Dual NRTI Backbone<br>• Drug: Current Regimen	Phase 4
69	Withdrawn	A Trial of Observed Long-acting, Anti-HIV Treatment With a Monoclonal CCR5 Antibody (PRO 140) as an Adjunct to a New, Optimized, Oral Antiretroviral Regimen in HIV-infected Injection Drug Users With Viral Rebound and Documented Poor Adherence	HIV	• Drug: PRO 140 targeting CCR5<br>• Drug: Placebo	Phase 2
70	Recruiting	A Randomised Placebo Controlled Trial of ART Plus Dual Long-acting HIV-specific Broadly Neutralising Antibodies (bNAbs).	HIV/AIDS and Infections	• Drug: Investigational Medicinal Product	Phase 2
71	Withdrawn	PRO 140 for Human Immunodeficiency Virus	HIV	• Biological: PRO 140 targeting CCR5	Phase 2
72	Not yet recruiting	The HIV Functional Cure Potential of UB-421 in ART Stabilized HIV-1 Patients	HIV-1-infection	• Biological: UB-421(25 mg/kg) Q2W<br>• Biological: UB-421(25 mg/kg) Q4W<br>CD4-targeting antibody	Phase 2
73	Active, not recruiting	The HIV Functional Cure Potential of UB-421 in ART Stabilized HIV-1 Patients	HIV-1 Infection	• Biological: UB-421(25 mg/kg) Q2W<br>• Biological: UB-421(25 mg/kg) Q4W<br>CD4-targeting antibody	Phase 2
74	Not yet recruiting	UB-421 Combine With Optimized Background Therapy Regimen in Multi-Drug Resistant HIV-1 Infection Patients	HIV-1 Infection	• Biological: UB-421<br>• Drug: Optimized background therapy (OBT)<br>CD4-targeting antibody	Phase 2
75	Completed	Dose Escalation Safety Study of TMB-365 in HIV-1 Infected Participants	HIV-1-infection	• Biological: TMB-365 targeting CD4 receptors	Phase 1
76	Active, not recruiting	A Pilot Trial of AVD and Brentuximab Vedotin (SGN-35) in the Treatment of Stage	HIV Infection	• Drug: brentuximab vedotin a CD30-specific mAbs<br>• Drug: Doxorubicin Hydrochloride	Phase 2

		III-IV HIV-associated Hodgkin Lymphoma	HIV-associated Hodgkin Lymphoma<br><br>Stage III Adult Hodgkin Lymphoma<br><br>Stage IV Adult Hodgkin Lymphoma	• Drug: Vinblastine<br>• Drug: Dacarbazine	
77	Recruiting	Effect of PCSK9 Inhibition on Cardiovascular Risk in Treated HIV Infection (EPIC-HIV Study)	Dyslipidemias<br><br>Cardiovascular Diseases<br><br>HIV Infections	• Drug: Alirocumab (PCSK9 inhibitory mAb)<br>• Other: Placebo	Phase 3
78	Recruiting	Albuvirtide and 3BNC117 as Long-Acting Maintenance Therapy in Virologically Suppressed Subjects	HIV-1-infection	• Drug: Albuvirtide targeting gp41<br>• Drug: 3BNC117 (targeting specific B cell receptors CD4 binding site)<br>• Drug: Baseline ART	Phase 2
79	Recruiting	Testing the Combination of the Anti-cancer Drugs XL184 (Cabozantinib) and Nivolumab in Patients With Advanced Cancer and HIV	Advanced Differentiated Thyroid Gland Carcinoma<br><br>Advanced Head and Neck Carcinoma<br><br>Advanced Hepatocellular Carcinoma<br><br>(and 100 more...)	• Drug: Cabozantinib S-malate<br>• Biological: Nivolumab (PD1-checkpoint inhibitor)	Phase 1
80	Withdrawn	the Study to Evaluate the Safety of UB-421 in Combination With Antiretroviral Therapy (ART) and the Efficacy in Reduction of HIV Viral Load and Proviral DNA as Compared to ART Alone in ART-experienced Viremic HIV-1 Patients	HIV-1 Infection	• Biological: UB-421 (CD4-targeting antibody)<br>• Other: Antiretroviral Therapy (ART)	Phase 2
81	Recruiting	Inducing Immune Quiescence the Genital Tract With ASA	Hiv	• Drug: ASA 81mg<br>• Other: Control Group<br>• Drug: ASA 325mg	Not Applicable
82	Recruiting	Nivolumab and Ipilimumab in Treating Patients With HIV Associated Relapsed or Refractory Classical Hodgkin Lymphoma or Solid Tumors That Are Metastatic or Cannot Be Removed by Surgery	Advanced Malignant Solid Neoplasm<br><br>Anal Carcinoma<br><br>HIV Infection<br><br>(and 6 more...)	• Biological: Ipilimumab inhibits CTLA4<br>• Biological: Nivolumab (PD1-checkpoint inhibitor)	Phase 1
83	Active, not recruiting	Brentuximab Vedotin and Combination Chemotherapy in Treating Patients With Stage II-IV HIV-Associated Hodgkin Lymphoma	AIDS-Related Hodgkin Lymphoma<br><br>Ann Arbor Stage II Hodgkin Lymphoma	• Drug: Brentuximab Vedotin a CD30-specific mAbs<br>• Drug: Dacarbazine<br>• Drug: Doxorubicin Hydrochloride	Phase 1<br>Phase 2

			Ann Arbor Stage IIA Hodgkin Lymphoma<br><br>(and 9 more...)	• (and 2 more...)	
84	Recruiting	Study of the Safety of Trogarzo™ Administered as an Undiluted "IV Push" or an Intramuscular Injection	HIV-1-infection	• Drug: ibalizumab-uiyk (CD4-**targeting** antibody)	Phase 3
85	Active, not recruiting<br>Has Results	Vorinostat and Combination Chemotherapy With Rituximab in Treating Patients With HIV-Related Diffuse Large B-Cell Non-Hodgkin Lymphoma or Other Aggressive B-Cell Lymphomas	AIDS-Related Plasmablastic Lymphoma<br><br>AIDS-Related Primary Effusion Lymphoma<br><br>Ann Arbor Stage I Diffuse Large B-Cell Lymphoma<br><br>(and 13 more...)	• Drug: Cyclophosphamide<br>• Drug: Doxorubicin Hydrochloride<br>• Drug: Etoposide<br>• (and 6 more...)<br><br>targeting CD20	Phase 1<br>Phase 2
86	Not yet recruiting	Safety, Immunogenicity, Efficacy of Ad26.Mos4.HIV, MVA-BN-HIV and PGT121, PGDM1400, and VRC07-523LS in HIV-1-Infected Adults	HIV<br><br>Acquired Immunodeficiency Syndrome<br><br>Immunologic Deficiency Syndrome, Acquired<br><br>(and 2 more...)	• Biological: Ad26.Mos4.HIV<br>• Biological: MVA-BN-HIV<br>• Biological: PGT121 targeting V3-glycan<br>• (and 2 more...)	Phase 1<br>Phase 2
87	Recruiting	Testing the Addition of an Experimental Medication MK-3475 (Pembrolizumab) to Usual Anti-Retroviral Medications in Patients With HIV and Cancer	AIDS-Related Non-Hodgkin Lymphoma<br><br>Classic Hodgkin Lymphoma<br><br>Clinical Stage III Cutaneous Melanoma AJCC v8<br><br>(and 27 more...)	• Other: Laboratory Biomarker Analysis<br>• Biological: Pembrolizumab (PD-1 inhibitor)	Phase 1
88	Recruiting	Therapy Adapted for High Risk and Low Risk HIV-Associated Anal Cancer	AIDS-Related Anal Carcinoma<br><br>Anal Basaloid Carcinoma<br><br>Anal Canal Cloacogenic Carcinoma<br><br>(and 20 more...)	• Drug: Capecitabine<br>• Drug: Fluorouracil<br>• Radiation: Intensity-Modulated Radiation Therapy<br>• (and 2 more...)	Phase 2
89	Completed<br>Has Results	Combination Chemotherapy and Rituximab in Treating Patients With HIV-Associated Stage I, Stage II, Stage III, or Stage IV Non-Hodgkin's Lymphoma	AIDS-related Diffuse Large Cell Lymphoma<br><br>AIDS-related Immunoblastic Large Cell Lymphoma	• Biological: rituximab targeting CD20<br>• Drug: etoposide<br>• Drug: doxorubicin hydrochloride<br>• (and 4 more...)	Phase 2

			AIDS-related Peripheral/Systemic Lymphoma<br><br>AIDS-related Small Noncleaved Cell Lymphoma		
90	Completed	Safety and Immune Response of BMS-936559 in HIV-Infected People Taking Combination Antiretroviral Therapy	HIV Infections	• Drug: BMS-936559 anti PDL-1<br>• Drug: Placebo for BMS-936559	Phase 1
91	Completed	Home Treatment of HIV-Infected Patients With Interleukin-2 With or Without a Tumor Necrosis Factor Antagonist	Acquired Immunodeficiency Syndrome<br><br>HIV Infection	• Drug: IL-2 plus Anti-TNF or Thalidomide	Phase 2
92	Recruiting<br>New	A Clinical Trial to Evaluate the Safety, Tolerability, and Pharmacokinetics of PGDM1400LS Alone and in Combination With VRC07-523LS and PGT121.414.LS in Healthy, HIV-uninfected Adult Participants	HIV Infections	• Drug: PGDM1400LS (5mg/kg, IV)<br>• Drug: PGDM1400LS (20mg/kg, IV)<br>• Drug: PGDM1400LS (20mg/kg, SC)<br>• (and 14 more...)<br><br>Targeting V1-V2 glycan	Phase 1
93	Recruiting	Evaluating the Safety and Immunogenicity of EnvSeq-1 and CH505 M5 gp120 Envs Adjuvanted With GLA-SE in Healthy, HIV-Uninfected Adults	HIV Infections	• Biological: CH505TF<br>• Biological: CH505w53<br>• Biological: CH505w78<br>• M5 gp120 Envs<br>• (and 3 more...)<br><br>M5 gp120 Envs	Phase 1
94	Unknown †	Rituximab Plus Combination Chemotherapy in Treating Patients With HIV-Related Non-Hodgkin's Lymphoma	Lymphoma	• Biological: bleomycin sulfate<br>• Biological: rituximab targeting CD20<br>• Drug: prednisolone<br>• Drug: vincristine sulfate	Phase 1
95	Completed<br>Has Results	Rituximab and Combination Chemotherapy in Treating Patients With Newly Diagnosed, HIV-Associated Burkitt's Lymphoma	Lymphoma	• Biological: filgrastim<br>• Biological: pegfilgrastim<br>• Biological: rituximab targeting CD20<br>• (and 10 more...)	Phase 2
96	Completed<br>Has Results	Combined Modality Therapy for Patients With With HIV and Stage I, Stage II, or Stage III Anal Cancer	Anal Cancer	• Biological: cetuximab targeting PD-L1<br>• Drug: cisplatin<br>• Drug: fluorouracil<br>• Radiation: radiation therapy	Phase 2
97	Active, not recruiting	VRC 603: A Phase I, Dose-Escalation Study of the Safety of AAV8-VRC07 (VRC-HIVAAV070-00-GT) Recombinant AAV Vector	HIV-1 Infected Adults With Controlled Viremia	• Genetic: VRC-HIVAAV070-00-GT (AAV8-VRC07)	Phase 1

		Expressing VRC07 HIV-1 Neutralizing Antibody in Antiretroviral -Treated, HIV-1 Infected Adults With Controlled Viremia.		(targeting specific B cell receptors CD4 binding site)	
98	Terminated	Rituximab in the Treatment of HIV Associated Multicentric Castleman Disease Dependent on Chemotherapy	HIV Infections<br><br>Giant Lymph Node Hyperplasia	• Drug: Rituximab targeting CD20	Phase 2
99	Terminated<br>Has Results	Bococizumab HIV Evaluation (B-HIVE) Study	Dyslipidemia<br><br>Cardiovascular Disease	• Drug: Bococizumab targeting PCSK9<br>• Drug: Placebo	Phase 3
100	Completed	Safety Study of Ibalizumab Subcutaneous Injection in Healthy Volunteers	Prevention of Infection With HIV-1	• Biological: ibalizumab targeting CD4 receptor (biologic/MAb) for SC Injection or placebo	Phase 1
101	Completed	Immunogenicity and Safety of Trivalent Influenza Vaccine in Non-pregnant HIV-infected Women	Influenza<br><br>Human Immunodeficiency Virus	• Biological: Trivalent Inactivated Influenza Vaccine	Phase 4
102	Completed	Once a Day Use of Lactobacillus Casei Shirota on HIV-infected Patients Infected Patients	HIV	• Dietary Supplement: Fermented Milk Drink Yakult 40	Phase 2
103	Recruiting	Ibrutinib, Rituximab, Etoposide, Prednisone, Vincristine Sulfate, Cyclophosphamide, and Doxorubicin Hydrochloride in Treating Patients With HIV-Positive Stage II-IV Diffuse Large B-Cell Lymphomas	AIDS-Related Lymphoma<br><br>Ann Arbor Stage II Diffuse Large B-Cell Lymphoma<br><br>Ann Arbor Stage III Diffuse Large B-Cell Lymphoma<br><br>Ann Arbor Stage IV Diffuse Large B-Cell Lymphoma	• Drug: Cyclophosphamide<br>• Drug: Doxorubicin Hydrochloride<br>• Drug: Etoposide<br>• (and 8 more...)	Phase 1
104	Terminated<br>Has Results	Phase I Study to Evaluate a Human Monoclonal Antibody (MAb) 10E8VLS Administered Alone or Concurrently With MAb VRC07-523LS Via Subcutaneous Injection in Healthy Adults	Healthy Adult Immune Response	• Biological: VRC-HIVMAB095-00-AB (10E8VLS) targeting gp41 MPER<br>• Biological: VRC-HIVMAB075-00-AB (VRC07-523LS) (targeting specific B cell receptors CD4 binding site)	Phase 1
105	Completed	Comparison of Two Methods in the Treatment of Cytomegalovirus of the Eyes in Patients With AIDS	Cytomegalovirus Retinitis<br><br>HIV Infections	• Drug: Sevirumab CCR5 targeting mAb<br>• Drug: Foscarnet sodium<br>• Drug: Ganciclovir	Phase 2
106	Completed	Combination Chemotherapy With or Without Oregovomab Followed by Stereotactic Body Radiation Therapy and	Pancreatic Adenocarcinoma	• Procedure: 4-Dimensional Computed Tomography<br>• Drug: Fluorouracil	Phase 2

	Has Results	Nelfinavir Mesylate in Treating Patients With Locally Advanced Pancreatic Cancer	Resectable Pancreatic Carcinoma<br><br>Stage I Pancreatic Cancer<br><br>(and 6 more...)	• Drug: Gemcitabine Hydrochloride<br>• (and 6 more...)	
107	Completed<br>Has Results	Doxorubicin Hydrochloride Liposome and Rituximab With Combination Chemotherapy in Treating Patients With Newly Diagnosed Burkitt's Lymphoma or Burkitt-Like Lymphoma	Lymphoma	• Drug: Regimen A<br>• Drug: Regimen B	Phase 2
108	Completed	Safety and Efficacy of an Antibody to CCR5 in Individuals With HIV Who Are Not Currently on Antiretroviral Therapy	HIV Infections	• Drug: CCR5mAb004	Phase 1
109	Completed<br>Has Results	Autologous Peripheral Stem Cell Transplant in Treating Patients With Non-Hodgkin's Lymphoma or Hodgkin's Lymphoma	Lymphoma	• Drug: carmustine<br>• Drug: cyclophosphamide<br>• Drug: etoposide<br>• (and 4 more...)	Phase 2
110	Completed	Immunogenicity and Safety of Trivalent Influenza Vaccine in Pregnant and Nonpregnant HIV Uninfected Women	Influenza	• Biological: Trivalent Influenza Vaccine	Phase 4
111	Active, not recruiting	A Study of Heterologous Vaccine Regimen of Adenovirus Serotype 26 Mosaic4 Human Immunodeficiency Virus(Ad26.Mos4.HIV), Adjuvanted Clade C gp140 and Mosaic gp140 to Prevent HIV-1 Infection Among Cis-gender Men and Transgender Individuals Who Have Sex With Cis-gender Men and/or Transgender Individuals	Healthy	• Biological: Ad26.Mos4.HIV<br>• Biological: Clade C and Mosaic gp140 HIV bivalent vaccine<br>• Biological: Placebo	Phase 3
112	Completed	Treatment Substitution With PRO 140 Monotherapy in Adult Subjects With HIV-1 Infection	HIV<br><br>Human Immunodeficiency Virus	• Drug: PRO 140 350mg weekly SQ injection. targeting CCR5	Phase 2
113	Active, not recruiting	An Extension of Protocol PRO 140_CD01 TS Study	HIV<br><br>Human Immunodeficiency Virus	• Drug: PRO 140 350mg weekly SQ injection targeting CCR5	Phase 2
114	Active, not recruiting<br>Has Results	Research In Viral Eradication of HIV Reservoirs	HIV	• Drug: Combination Antiretroviral Therapy (cART)<br>• Drug: Raltegravir<br>• Drug: Vorinostat	Phase 2

				• (and 2 more...)	
115	Unknown †	Safety and Pharmacokinetics of the Combination Broadly Neutralizing Antibodies, 3BNC117-LS-J and 10-1074-LS-J, in Healthy American and African Adults	HIV-1-infection	• Biological: 3BNC117-LS-J (targeting specific B cell receptors CD4 binding site)<br>• Biological: 10-1074-LS-J targeting V3 glycan<br>• Biological: Combination 3BNC117-LS-J and 10-1074-LS-J<br>• (and 4 more...)	Phase 1<br>Phase 2
116	Completed	Vandetanib and Bevacizumab in Treating Patients With Advanced Solid Tumors or Lymphoma	Lung Cancer<br>Lymphoma<br>Lymphoproliferative Disorder<br>(and 2 more...)	• Biological: bevacizumab anti-VEGF monoclonal antibody<br>• Drug: vandetanib<br>• Other: laboratory biomarker analysis<br>• Other: pharmacological study	Phase 1
	**Ebolaviruses** a member of Filoviridae (-ssRNA viruses-Grroup V)				
1	Not yet recruiting	Prophylaxis Vaccine Antibodies Ebola	Ebola Virus Disease	• Drug: ansuvimab targeting B cell maturation antigen<br>• Biological: Ervebo targeting The EBOV surface glycoprotein	Phase 2
2	Recruiting	An Open Study of the Safety and Pharmacokinetics of a Drug for the Emergency Prevention of Ebola Virus Disease	Ebola Virus Disease	• Drug: Gamezumab	Phase 1
3	Completed	An Open Study of the Safety and Pharmacokinetics of a Medicinal Product for Emergency Prevention of Ebola	Healthy	• Biological: GamEMab	Phase 1
4	Completed	Study to Evaluate the Immunogenicity and Safety of an Ebola Virus (EBOV) Glycoprotein (GP) Vaccine in Healthy Subjects	Ebola	• Biological: Base Dose EBOV GP Vaccine<br>• Biological: 2x Base Dose EBOV GP Vaccine<br>• Biological: 4x Base Dose EBOV GP Vaccine<br>• (and 3 more...)	Phase 1
5	Not yet recruiting<br>New	Pilot Study Evaluating the Impact of Delay Between Administration of Inmazeb Administration and Vaccination by Ervebo on Vaccine Immune Response on Healthy Volunteers	Ebola Virus Disease	• Biological: Ervebo targeting The EBOV surface glycoprotein<br>• Drug: Inmazeb	Phase 2
6	Withdrawn	Safety and Pharmacokinetics of a Single ZMappTM Administration in Healthy Adult Volunteers	Healthy	• Drug: ZMAPP<br>**GP-targeting monoclonal antibodies**	Phase 1

7	Completed<br>Has Results	Safety and Pharmacokinetics of a Human Monoclonal Antibody, VRC-EBOMAB092-00-AB (MAb114), Administered Intravenously to Healthy Adults	Healthy Adult Immune Responses to Vaccine	• Biological: VRC-EBOMAB092-00-AB (MAb114)<br><br>**GP-targeting monoclonal antibodies**	Phase 1
	**Influenza viruses** a member of *Orthomyxoviridae* family (-ssRNA viruses-Grroup V)				
1	Completed	Safety Study of Anti-Influenza Virus Monoclonal Antibody to Treat Influenza	Influenza, Human	• Biological: TCN-032 (Anti-M2e mAbs<br>• Biological: Placebo	Phase 1
2	Unknown †	Influenza Virus Challenge Study to Test Monoclonal Antibody TCN-032 as a Treatment for Influenza	Influenza	• Biological: TCN-032 (Anti-M2e mAb<br><br>• Biological: Placebo (saline)	Phase 2
3	Completed	Assessment of CR8020, a Monoclonal Antibody Against Influenza A Viruses	Influenza	• Biological: 2 mg/kg CR8020<br>• Biological: 5 mg/kg CR8020<br>• Biological: 15 mg/kg CR8020<br>• (and 3 more...)<br><br>bind the stem region of the HA molecule	Phase 1
4	Completed	Assessment of CR6261, a Monoclonal Antibody Against the Influenza A Virus	Influenza	• Biological: CR6261 2 mg/kg<br>• Biological: CR6261 5 mg/kg<br>• Biological: CR6261 15 mg/kg<br>• (and 3 more...)<br><br>bind the stem region of the HA molecule	Phase 1
5	Unknown †	Safety and Pharmacokinetics Study of Human Monoclonal Antibody (FGI-101-1A6)	Influenza	• Drug: FGI-101-1A6 targeting a universally conserved epitope of TSG-*101*	Phase 1
6	Withdrawn	Assessment of Efficacy of CR8020 and CR6261, Monoclonal Antibodies, Against Influenza Infection	Influenza	• Biological: CR8020<br>• Biological: CR6261<br>• Biological: Placebo<br><br>(the epitope is largely distinct from the site recognized by CR6261/F10, and is even lower down on the HA stem)	Phase 2
7	Completed	Evaluation of Human Immune Responses to Influenza Virus Vaccination in Healthy Volunteers	Influenza<br><br>Influenza Immunisation	• Biological: Influenza Virus Vaccine Inactivated	Phase 4
8	Completed	Evaluation of the Protective Efficacy and Safety of CR8020 in an Influenza Challenge	Influenza	• Biological: CR8020 (the epitope is largely distinct from the site recognized by CR6261/F10, and is even lower down on the HA stem)<br>• Biological: Placebo	Phase 2

9	Completed	Immunogenicity and Safety of Trivalent Influenza Vaccine in Non-pregnant HIV-infected Women	Influenza<br><br>Human Immunodeficiency Virus	• Biological: Trivalent Inactivated Influenza Vaccine	Phase 4
10	Withdrawn	Evaluation of the Safety and Efficacy of a Monoclonal Antibody to Treat Influenza	Influenza	• Drug: Oseltamivir<br>• Biological: MEDI8852 binds a highly conserved influenza A hemagglutinin stalk epitope<br>• Drug: Placebo	Phase 2
11	Completed	A Study of VIS410 to Assess Safety and Pharmacokinetics	Influenza	• Drug: VIS410 (binds a highly conserved influenza A hemagglutinin stalk epitope)<br>• Drug: Placebo	Phase 1
12	Completed<br>Has Results	A Study of MHAA4549A as Monotherapy for Acute Uncomplicated Seasonal Influenza A in Otherwise Healthy Adults	Influenza A	• Drug: MHAA4549A target highly conserved epitope on the stalk of influenza A hemagglutinin<br>• Drug: Placebo	Phase 2
13	Not yet recruiting	Impact of Inactivated Trivalent Influenza Vaccine on NSCLC Patients Receiving PD-1 / PD-L1 Inhibitors	Non-Small Cell Lung Cancer<br><br>Influenza Vaccine	• Drug: PD-1/PD-L1 inhibitors<br>• Biological: Inactivated trivalent influenza vaccine	•
14	Completed<br>Has Results	Efficacy and Safety of CR6261 in an H1N1 Influenza Healthy Volunteer Human Challenge Model	H1N1 Influenza Healthy Volunteers	• Biological: CR6261 V_H1-69 antibodies bind the stem region of the HA molecule<br>• Biological: Placebo	Phase 2
15	Completed	Phase 1 Placebo-controlled,Dose-escalation Study to Evaluate the Safety and Pharmacokinetics of MEDI8852 in Adults	Influenza	• Drug: MEDI8852 binds a highly conserved influenza A hemagglutinin stalk epitope<br>• Drug: Placebo	Phase 1
16	Completed<br>Has Results	A Phase 2a to Evaluate the Safety of MEDI8852 in Adults With Uncomplicated Influenza	Influenza	• Drug: Oseltamivir<br>• Drug: MEDI8852 binds a highly conserved influenza A hemagglutinin stalk epitope<br>• Drug: Placebo	Phase 1<br>Phase 2
17	Withdrawn	Dose Ranging Study to Evaluate the Efficacy and Safety of MEDI8852 in Adults Who Are Hospitalized With Type A Influenza.	Influenza - Type A Strains	• Drug: Oseltamivir<br>• Drug: MEDI8852 binds a highly conserved influenza A hemagglutinin stalk epitope<br>• Drug: Placebo	Phase 2
18	Completed	Evaluation of the Safety and Immunogenicity of a Recombinant Trivalent Nanoparticle Influenza Vaccine With Matrix M-1 Adjuvant (NanoFlu)	Influenza	• Biological: NanoFlu<br>• Biological: Fluzone HD - Day 0<br>• Biological: Fluzone HD - Day 21<br>• Other: Saline - Day 21	Phase 1<br>Phase 2

19	Completed Has Results	Safety and Immunogenicity of Two Doses of a Tetravalent Influenza Vaccine in Adults Aged 18 Years and Above	Influenza	• Biological: MF59-eTIV-H5N1+ placebo /pandemic influenza vaccine<br>• Biological: Pandemic influenza vaccine + placebo /MF59-eTIV-H5N1<br>• Biological: Pandemic influenza vaccine + seasonal influenza vaccine /pandemic influenza vaccine<br>• (and 3 more...)	Phase 2
20	Completed Has Results	Safety and Immunogenicity of a Live-attenuated Universal Flu Vaccine Followed by an Inactivated Universal Flu Vaccine	Influenza<br><br>Vaccine	• Biological: cH8/1N1 LAIV<br>• Biological: AS03-adjuvanted cH5/1N1 IIV<br>• Biological: cH5/1N1 IIV<br>• (and 3 more...)	Phase 1
21	Completed	Immunogenicity and Safety of Trivalent Influenza Vaccine in Pregnant and Nonpregnant HIV Uninfected Women	Influenza	• Biological: Trivalent Influenza Vaccine	Phase 4
	**SARS-CoV-2 virus** a member of Retroviridae (+ssRNA virus Group IV)				
1	Recruiting New	TURN-COVID Biobank: The Dutch Cohort Study for the Evaluation of the Use of Neutralizing Monoclonal Antibodies and Other Antiviral Agents Against SARS-CoV-2	COVID-19	• Drug: casirivimab ***target* the receptor-binding domain of the spike protein of SARS** with imdevimab **bind to non-overlapping epitopes of the RBD**<br>• Drug: sotrovimab (targets an epitope in the RBD of the spike protein that is conserved between SARS-CoV and SARS-CoV-2)<br>• Drug: molnupiravir	•
2	Withdrawn	A Safety and Efficacy Study of Human Monoclonal Antibodies, BRII-196 and BRII-198 for the Treatment of Patients With COVID-19	COVID-19	• Drug: BRII-196 and BRII-198 targets an epitope in the RBD of the spike protein<br>• Drug: Placebo	Phase 2
3	Recruiting	Tolerability,Safety,Pharmacokinetic Profile and Immunogenicity of a Recombinant Humanized Anti-SARS-CoV-2 Monoclonal Antibody (JS016) for Injection in Chinese Health Subjects	COVID-19; and High Infection Risk of SARS-CoV-2	• Combination Product: JS016 (anti-SARS-CoV-2 monoclonal antibody) *target* the HR1 domain	Phase 1
4	Active, not recruiting	Study to Evaluate the Safety and Concentrations of Monoclonal Antibody Against Virus That Causes COVID-19 Disease.	COVID-19 Virus Disease	• Biological: MAD0004J08 targeting the spike protein of SARS-CoV-2<br>• Other: Placebo	Phase 1
5	Recruiting	JS016 (Anti-SARS-CoV-2 Monoclonal Antibody)With Mild and Moderate COVID-19 or SARS-CoV-2	COVID-19	• Biological: Recombinant Human Anti-SARS-CoV-2 Monoclonal Antibody(25mg/kg;50mg/kg;100 mg/kg)	Phase 1 Phase 2

		Asymptomatic Infection Subects		• Drug: Placebo<br><br>targeting the spike protein of SARS-CoV-2	
6	Recruiting<br>New	Non-inferiority Trial on Monoclonal Antibodies in COVID-19	COVID-19	• Drug: Bamlanivimab Etesevimab that bind to different, but overlapping, epitopes in the spike protein RBD of SARS-CoV-2<br>• Drug: Sotrovimab (targets an epitope in the RBD of the spike protein)<br>• Drug: Casirivimab-Imdevimab *target* the receptor-binding domain of the spike protein of SARS	Phase 3
7	Complete d	COVID-19 : Neutralizing Human Monoclonal Antibodies Against SARS-Cov-2	COVID	• Other: Blood sample	•
8	Recruiting	Study to Select the Dose and Evaluate Safety and Efficacy of Monoclonal Antibody in Adult With Recently Diagnosed Asymptomatic to Moderately Severe COVID-19.	Covid19	• Drug: MAD0004J08 targeting the spike protein of SARS-CoV-2<br>• Other: Placebo	Phase 2<br>Phase 3
9	Recruiting	Use of Monoclonal Antibodies for the Treatment of Mild to Moderate COVID-19 in Non-Hospitalized Setting	Covid19	• Biological: BAMLANIVIMAB that bind to different, but overlapping, epitopes in the spike protein RBD of SARS-CoV-2<br>• Biological: CASIRIVIMAB<br>• Biological: IMDEVIMAB<br><br>*target* the receptor-binding domain of the spike protein of SARS	Phase 2
10	Complete d	A Study of Human Monoclonal Antibodies, BRII-196 and BRII-198	COVID-19	• Drug: BRII-196 and BRII-198 (targets an epitope in the RBD of the spike protein)<br>• Drug: Placebo	Phase 2
11	Complete d	SARS-CoV-2-Neutralizing Monoclonal COVID-19 Antibody DZIF-10c by Infusion	SARS-CoV-2 Infection	• Biological: DZIF-10c binds the receptor-binding domain of the SARS-CoV-2 spike protein<br>• Other: Placebo	Phase 1<br>Phase 2
12	Not yet recruiting<br>New	Pre-exposure Prophylaxis of SARS-CoV-2 Infection (COVID-19) by Monoclonal Antibodies With Early Access Authorization in Immunocompromised Patients. A Prospective Cohort.	COVID-19<br><br>Immunocompromised Host		

13	Completed	SARS-CoV-2-Neutralizing Monoclonal COVID-19 Antibody DZIF-10c by Inhalation	SARS-CoV-2 Infection	• Biological: DZIF-10c binds the receptor-binding domain of the SARS-CoV-2 spike protein<br>• Drug: Placebo	Phase 1<br>Phase 2
14	Recruiting	Safety, Tolerability, and Efficacy of Anti-Spike (S) SARS-CoV-2 Monoclonal Antibodies for the Treatment of Ambulatory Adult and Pediatric Patients With COVID-19	COVID-19	• Drug: casirivimab+imdevimab combination therapy bind to nonoverlapping epitopes of the spike protein RBD of SARS-CoV-2.	Phase 3
15	Recruiting	COVID-19 Administration of Single-Dose Subcutaneous Anti- Spike(s) SARS-CoV-2 Monoclonal Antibodies Casirivimab and Imdevimab in High-Risk Pediatric Participants Under 12 Years of Age	COVID-19	• Drug: casirivimab+imdevimab bind to nonoverlapping epitopes of the spike protein RBD of SARS-CoV-2.	Phase 2
16	Not yet recruiting	COVID-19 Study of Pharmacokinetics, Safety, Tolerability, and Efficacy of Intravenous Anti-Spike(s) SARS-CoV-2 Monoclonal Antibodies (Casirivimab+Imdevimab) for the Treatment of Pediatric Patients Hospitalized Due to COVID-19	COVID-19	• Drug: casirivimab+imdevimab bind to nonoverlapping epitopes of the spike protein RBD of SARS-CoV-2.	Phase 1
17	Completed	Safety, Tolerability, and Efficacy of Anti-Spike (S) SARS-CoV-2 Monoclonal Antibodies for Hospitalized Adult Patients With COVID-19	COVID-19	• Drug: REGN10933+REGN10987 combination therapy *target* different epitopes on the SARS-CoV-2 spike protein.<br>• Drug: Placebo	Phase 1<br>Phase 2
18	Completed	Safety, Tolerability and Pharmacokinetics of SCTA01, an Anti-SARS-CoV-2 Monoclonal Antibody, in Healthy Chinese Subjects	Coronavirus Disease 2019(COVID-19)	• Biological: SCTA01 target the SARS-CoV-2 spike protein<br>• Other: Placebo	Phase 1
19	Not yet recruiting	Impact of Monoclonal Antibody Treatment on Post-Acute COVID-19 Syndrome	Post-acute COVID-19 (PACS), or "Long COVID" Syndrome	• Other: Surveys	•
20	Recruiting	The Efficacy and Safety of SCTA01 in Hospitalized Patients With Severe COVID-19	Covid19	• Drug: SCTA01 target the SARS-CoV-2 spike protein<br>• Other: Placebo	Phase 2<br>Phase 3
21	Recruiting	Insight Into the UAE Experience With Monoclonal Antibodies (Sotrovimab)	COVID-19 Respiratory Infection		

22	Recruiting	A Phase 1 Study of Human Monoclonal Antibodies, BRII-196 and BRII-198	COVID-19	• Drug: BRII-196 and BRII-198 targets an epitope in the RBD of the spike protein<br>• Drug: Placebo	Phase 1
23	Recruiting	Finding Treatments for COVID-19: A Trial of Antiviral Pharmacodynamics in Early Symptomatic COVID-19 (PLATCOV)	COVID-19	• Drug: Favipiravir<br>• Drug: Monoclonal antibodies<br>• Drug: Ivermectin inhibits nuclear transport mechanisms mediated by importins<br>• (and 2 more...)	Phase 2
24	Completed	Safety, Tolerability, and Pharmacokinetics Study of Human Monoclonal Antibody BRII-198	COVID-19	• Drug: BRII-198 targets an epitope in the RBD of the spike protein<br>• Drug: Placebo	Phase 1
25	Completed	Safety, Tolerability, and Pharmacokinetics Study of Human Monoclonal Antibody BRII-196	COVID-19	• Drug: BRII-196 targets an epitope in the RBD of the spike protein<br>• Drug: Placebo	Phase 1
26	Completed	Study of CPI-006 as Immunotherapy for Hospitalized COVID-19 Patients	COVID-19	• Drug: CPI-006 an Anti-CD73 Antibody<br>• Other: Standard of Care	Phase 1
27	No longer available	Compassionate Use Open-Label Anti-CD14 Treatment in Patients With SARS-CoV-2 (COVID-19)	COVID<br>ARDS, Human<br>Ards<br>SARS-CoV2	• Biological: IC14, a monoclonal antibody against CD14	•
28	Recruiting	UPMC OPTIMISE-C19 Trial, a COVID-19 Study	Covid19	• Biological: Lilly Bamlanivimab<br>• Biological: Regeneron Casirivimab + Imdevimab *target* the receptor-binding domain of the spike protein of SARS<br>• Biological: Lilly Bamlanivimab + Etesevimab that bind to different, but overlapping, epitopes in the spike protein RBD of SARS-CoV-2<br>• Biological: Sotrovimab (targets an epitope in the RBD of the spike protein that is conserved between SARS-CoV and SARS-CoV-2)	Phase 3
29	Completed<br>Has Results	Treatment With CSL312 in Adults With Coronavirus Disease 2019 (COVID-19)	Coronavirus Disease 2019 (COVID-19)	• Biological: Garadacimab, Factor XIIa Antagonist Monoclonal Antibody<br>• Drug: Placebo	Phase 2

30	Active, not recruiting	Intramuscular VIR-7831 (Sotrovimab) for Mild/Moderate COVID-19	Covid19	• Biological: VIR-7831 (targets an epitope in the RBD of the spike protein that is conserved between SARS-CoV and SARS-CoV-2)	Phase 3
31	Active, not recruiting	Immune Responses to COVID-19; Isolation of Neutralizing Antibodies for Therapeutics and Vaccine.	SARS-CoV 2	• Other: Blood sampling	•
32	Recruiting	Efficacy and Safety of JS016 in Patients With SARS-CoV-2 Infection (COVID-19)	COVID-19<br><br>SARS-CoV-2	• Drug: JS016 *target* the HR1 domain	Phase 2
33	Recruiting	COVID-19 International Drug Pregnancy Registry	Covid19	• Drug: Monoclonal antibody	
34	Recruiting	Use of nMoABs for the Treatment of COVID-19 in Patients With HM.	Covid19<br><br>Hematological Malignancy		
35	Completed	A Study of Immune System Proteins in Participants With Mild to Moderate COVID-19 Illness	COVID-19	• Drug: LY3819253 anti-spike mAbs<br>• Drug: LY3832479 anti-spike mAbs<br>• Drug: Placebo<br>• (and 2 more...)	Phase 2
36	Not yet recruiting<br>New	COVID-19 Study Assessing the Safety and Tolerability of Co-Formulated Anti-Spike (S) SARS-CoV-2 Monoclonal Antibodies (Casirivimab+Imdevimab) in Adult Volunteers	Healthy<br><br>Chronic Stable Illness	• Drug: Casirivimab+Imdevimab<br><br>*target* the receptor-binding domain of the spike protein of SARS	Phase 1
37	Completed	Study Assessing the Safety, Tolerability, Pharmacokinetics, and Immunogenicity of Repeated Subcutaneous Doses of Anti-Spike (S) SARS-CoV-2 Monoclonal Antibodies (REGN10933+REGN10987) in Adult Volunteers as Related to COVID-19	Healthy<br><br>Chronic Stable Illness	• Drug: REGN10933+REGN10987 *target* different epitopes on the SARS-CoV-2 spike protein.<br>• Drug: Placebo	Phase 1
38	Recruiting	A Study to Evaluate Efficacy and Safety of Casirivimab+Imdevimab (Monoclonal Antibodies) for Prevention of COVID-19 in Immunocompromised Adolescents and Adults	Immunocompromised	• Drug: casirivimab+imdevimab *target* the receptor-binding domain of the spike protein of SARS<br>• Drug: Placebo	Phase 3
39	Not yet recruiting	The Safety and Efficacy of SCTA01 Against COVID-19 in	Covid19	• Biological: SCTA01 target the SARS-CoV-2 spike protein	Phase 2<br>Phase 3

		Patients Admitted to High Dependence or Intensive Care		• Biological: SCTA01 Placebo	
40	Not yet recruiting	To Evaluate SCTA01 Treatment of High-risk Outpatients With COVID-19	COVID-19<br><br>SARS-CoV-2	• Drug: SCTA01 target the SARS-CoV-2 spike protein<br>• Other: Placebo	Phase 2<br>Phase 3
41	Completed	COVID-19 Study Assessing the Efficacy and Safety of Anti-Spike SARS CoV-2 Monoclonal Antibodies for Prevention of SARS CoV-2 Infection Asymptomatic in Healthy Adults and Adolescents Who Are Household Contacts to an Individual With a Positive SARS-CoV-2 RT-PCR Assay	Healthy Participants	• Drug: REGN10933 + REGN10987 *target* different epitopes on the SARS-CoV-2 spike protein.<br>• Drug: Placebo	Phase 3
42	Not yet recruiting	Study to Evaluate the Safety and Efficacy of a Monoclonal Antibody Cocktail for the Prevention of COVID-19	COVID-19	• Drug: ADM03820 incorporating mAbs that target non-overlapping regions of the RBD<br>• Other: Placebo	Phase 2<br>Phase 3
43	Suspended	Treatment of Nosocomial COVID-19	Covid19<br><br>Nosocomial Infection<br><br>SARS-CoV2 Infection	• Drug: Anti-SARS-CoV-2 mAb	Phase 4
44	Not yet recruiting	Pharmacokinetics of Sotrovimab as Pre-exposure Prophylaxis for COVID-19 in Hematopoietic Stem Cell Transplant Recipients, COVIDMAB Study	COVID-19 Infection<br><br>Hematopoietic and Lymphoid Cell Neoplasm<br><br>Malignant Solid Neoplasm	• Other: Questionnaire Administration<br>• Biological: Sotrovimab (targets an epitope in the RBD of the spike protein that is conserved between SARS-CoV and SARS-CoV-2)	Phase 1
45	Recruiting	COVID-19 and Anti-CD14 Treatment Trial	Coronavirus Disease 2019 (COVID-19)<br><br>Severe Acute Respiratory Syndrome Coronavirus 2 (SARS-CoV-2)	• Biological: anti-CD14<br>• Other: Placebo<br>• Drug: remdesivir	Phase 2
46	No longer available	COVID-19 Soliris Expanded Access Protocol	Covid19	• Biological: Eculizumab targets complement **protein C5**	•
47	Recruiting	Study of TJ003234 (Anti-GM-CSF Monoclonal Antibody) in Subjects With Severe Coronavirus Disease 2019 (COVID-19)	Coronavirus Disease 2019 COVID-19	• Drug: TJ003234 Anti-GM-CSF Monoclonal Antibody<br>• Drug: Placebo	Phase 2<br>Phase 3
48	Active, not recruiting	Phase III Double-blind, Placebo-controlled Study of AZD7442 for Post- Exposure	COVID-19	• Drug: AZD7442 *target* the HR1 domain	Phase 3

		Prophylaxis of COVID-19 in Adults		• Drug: Placebo	
49	Completed	Efficacy and Safety of hzVSF-v13 in Moderate to Severe Patients With COVID-19 Pneumonia	COVID-19	• Drug: hzVSF-v13 humanized virus suppressing factor<br>• Drug: Placebo (Normal saline solution)	Phase 2
50	Active, not recruiting	Phase III Double-blind, Placebo-controlled Study of AZD7442 for Pre-exposure Prophylaxis of COVID-19 in Adult.	COVID-19	• Drug: AZD7442 *target* the HR1 domain<br>• Drug: Placebo	Phase 3
51	Terminated	Study of the Efficacy and Safety of Intravenous Pamrevlumab, in Hospitalized Participants With Acute COVID-19 Disease	COVID-19	• Drug: Pamrevlumab an anti-connective tissue growth factor<br>• Drug: Placebo	Phase 2
52	Recruiting	Efficacy and Safety of hzVSFv13 in Patients With COVID-19 Pneumonia	COVID-19	• Drug: hzVSF-v13 humanized virus suppressing factor<br>• Drug: Placebo (Normal saline solution)	Phase 2
53	Completed	VIR-7831 for the Early Treatment of COVID-19 in Outpatients	Covid19	• Biological: VIR-7831 anti-spike mAbs<br>• Drug: Placebo	Phase 2<br>Phase 3
54	Withdrawn	A Study to Evaluate Efficacy and Safety of the Combination of SCTA01 & SCTA01C in Outpatients With COVID-19	COVID-19	• Drug: SCTA01 and SCTA01C target the SARS-CoV-2 spike protein<br>• Drug: Placebo	Phase 1<br>Phase 2
55	Terminated	Efficacy and Safety of Emapalumab and Anakinra in Reducing Hyperinflammation and Respiratory Distress in Patients With COVID-19 Infection.	SARS-CoV-2	• Biological: Emapalumab targeting **IFN-γ**<br>• Biological: Anakinra	Phase 2<br>Phase 3
56	Withdrawn	Study of TB006 in Outpatient Patients With Mild to Moderate COVID-19	Covid19	• Drug: TB006<br>• Other: Placebo	Phase 1
57	Recruiting	Study of Monoclonal Antibody Cocktail Being Tested for the Prevention of COVID-19	SARS-CoV-2	• Drug: ADM03820 incorporating mAbs that target non-overlapping regions of the RBD<br>• Other: Placebo	Phase 1
58	Active, not recruiting	Phase 3 Study to Evaluate Efficacy and Safety of Lenzilumab in Patients With COVID-19	Coronavirus Disease 2019 (COVID-19) Pneumonia	• Biological: Lenzilumab a GM-CSF antagonist<br>• Drug: Standard of Care	Phase 3
59	Completed	Acquiring Convalescent Specimens for COVID-19 Antibodies	COVID-19<br>Coronavirus Infection	• Procedure: Blood draw	•

			Corona Virus Infection		
60	Completed	Safety of TY027, a Treatment for COVID-19, in Humans	Coronavirus Disease-2019 (COVID-19)	• Biological: TY027 targeting spike protein<br>• Other: 0.9% Saline	Phase 1
61	Active, not recruiting	RU Anti-SARS-CoV-2 (COVID-19) mAbs in Healthy Volunteers	Covid19	• Biological: C144-LS and C-135-LS *antibodies* that *target* the RBD	Phase 1
62	Completed	Efficacy and Safety of hzVSF-v13 in Patients With COVID-19 Pneumonia	COVID-19	• Drug: hzVSF-v13 humanized virus suppressing factor<br>• Drug: Placebo (Normal saline solution)	Phase 2
63	Recruiting	Monitoring of COVID-19 Vaccine Response in Organ Transplant Patients	Covid19<br><br>Kidney Transplantation		
64	Not yet recruiting	Evaluate Safety and Pharmacokinetics of HLX70 in Healthy Adult Volunteers	COVID 19	• Drug: HLX70 peptides *target* the HR1 domain<br>• Other: Placebo	Phase 1
65	Not yet recruiting	Immune Response After SARS-CoV-2 Vaccination in a Context of Non-Hodgkin Lymphoma	Non-hodgkin Lymphoma,B Cell<br><br>Vaccination Failure	• Other: Immunological analyses	Not Applicable
66	Not yet recruiting	Observational Study on the Use of Ivermectin as an Outpatient Treatment Option for COVID-19	Covid19<br><br>COVID-19 Pneumonia<br><br>COVID-19 Respiratory Infection<br><br>COVID-19 Acute Bronchitis	• Drug: Ivermectin inhibits nuclear transport mechanisms mediated by importins	•
67	Recruiting	Leronlimab in Moderately Ill Patients With COVID-19 Pneumonia	COVID-19 Pneumonia	• Drug: Leronlimab bind CCR5<br>• Drug: Placebo	Phase 3
68	Recruiting	Leronlimab in Patients With Coronavirus Disease 2019 (COVID-19) With Need for Mechanical Ventilation or Extracorporeal Membrane Oxygenation	COVID-19 Pneumonia	• Drug: Leronlimab bind CCR5<br>• Drug: Placebo	Phase 3
69	Not yet recruiting	Transplantation of Deceased Donors With COVID-19 Into COVID-19 Negative Recipients Utilizing Casirivimab and Imdevimab Antibody Cocktail	COVID-19<br><br>Organ Transplant	• Drug: Casirivimab and Imdevimab Antibody Cocktail *target* the receptor-binding domain of the spike protein of SARS	Phase 1
70	Recruiting	Efficacy and Safety of TY027, a Treatment for COVID-19, in Humans	Coronavirus Disease-2019 (COVID-19)	• Biological: TY027 targeting spike protein<br>• Other: 0.9% saline	Phase 3

71	Completed<br>Has Results	A Study to Assess the Efficacy and Safety of Gimsilumab in Subjects With Lung Injury or Acute Respiratory Distress Syndrome Secondary to COVID-19 (BREATHE)	COVID-19	• Drug: Gimsilumab Anti-GM-CSF monoclonal *antibody*<br>• Drug: Placebo	Phase 2
72	Recruiting	Study on the Use of Sarilumab in Patients With COVID-19 Infection	COVID19	• Drug: Sarilumab Prefilled Syringe *targeted* against the alpha subunit of the IL-6 receptor complex	Early Phase 1
73	Suspended	Study of Immune Modulatory Drugs and Other Treatments in COVID-19 Patients: Sarilumab, Azithromycin, Hydroxychloroquine Trial - CORIMUNO-19 - VIRO	COVID19<br>SARS-CoV-2 Infection	• Drug: Sarilumab *targeted* against the alpha subunit of the IL-6 receptor complex<br>• Drug: Azithromycin<br>• Drug: Hydroxychloroquine	Phase 2<br>Phase 3
74	Completed	Efficacy of Subcutaneous Sarilumab in Hospitalised Patients With Moderate-severe COVID-19 Infection (SARCOVID)	Covid-19	• Drug: Sarilumab *targeted* against the alpha subunit of the IL-6 receptor complex<br>• Other: Standar of care	Phase 2
75	Withdrawn	Clinical and Immunological Evolution of Covid-19 Occurring in a Context of Non-Hodgkin Lymphoma	B-cell Lymphoma<br>Covid19		
76	Not yet recruiting	Exploratory Regimen of Basiliximab for Treatment of Pulmonary Cytokine Storm in SARS-CoV-2 Hospitalized Adult Patients	SARS-CoV-2 Acute Respiratory Disease<br>SARS-CoV2 Infection<br>Cytokine Storm	• Drug: Basiliximab Injection *targeted* against the alpha subunit of the IL-6 receptor complex<br>• Drug: Placebo	Phase 2
77	Recruiting	Bevacizumab in Severe or Critically Severe Patients With COVID-19 Pneumonia-RCT	COVID-19 Pneumonia	• Drug: Bevacizumab anti-VEGF monoclonal antibody	Not Applicable
78	Completed<br>New	Study of the Outcomes of Olokizumab Therapy in Hospitalized Patients With SARS-CoV-2 (COVID-19) Infection	COVID-19	• Drug: Olokizumab anti-IL-6 monoclonal *antibody*<br>• Drug: Standard therapy	•
79	Terminated	RAPA-501-Allo Therapy of COVID-19-ARDS	Severe COVID-19 Disease	• Biological: RAPA-501-Allo off-the-shelf Therapy of COVID-19<br>• Other: Placebo	Phase 1<br>Phase 2
80	Completed	Study to Assess Adverse Events and How Intravenous (IV) ABBV-47D11 and IV ABBV-2B04 Given Alone and in Combination Moves Through the Body of Adult Participants With Coronavirus Disease 2019 (COVID-19)	CoronaVirus Disease-2019 (COVID-19)	• Drug: ABBV-47D11 target the S glycoprotein<br>• Drug: Placebo for ABBV-47D11<br>• Drug: ABBV-2B04 S glycoprotein<br>• Drug: Placebo for ABBV-2B04	Phase 1

81	Completed	A Study of AK119 (Anti-CD73 Antibody), a Treatment for COVID-19, in Healthy Subjects	Coronavirus Disease 2019 (COVID-19)	• Drug: AK119 anti CD-73<br>• Drug: Placebo	Phase 1
82	Withdrawn	Study to Evaluate the Safety, Pharmacokinetics and Efficacy of STI-2020 (COVI-AMG™) in Outpatients With COVID-19	Covid19	• Biological: COVI-AMG targeting IL33 R<br>• Drug: Placebo	Phase 1<br>Phase 2
83	Not yet recruiting	Study to Evaluate the Safety and Efficacy of a Single Dose of STI-2020 (COVI-AMG™) to Treat COVID-19	Covid19	• Biological: COVI-AMG targeting IL33 R<br>• Drug: Placebo	Phase 1<br>Phase 2
84	Recruiting	Study to Evaluate a Single Dose of STI-2020 (COVI-AMG™) in Hospitalized Adults With COVID-19	Covid19	• Biological: COVI-AMG targeting IL33 R<br>• Drug: Placebo	Phase 2
85	Recruiting	Study to Evaluate a Single Dose of STI-2020 (COVI-AMG™) in Adults With Mild COVID-19 Symptoms	Covid19	• Biological: COVI-AMG targeting IL33 R<br>• Drug: Placebo	Phase 2
86	Not yet recruiting	Randomized Study to Evaluate Intranasal Dose of STI-2099 (COVI-DROPS™) in Outpatient Adults With Mild COVID-19 Infection	COVID-19	• Biological: COVI-DROPS anti-SARS-CoV-2 spike neutralizing antibodies<br>• Drug: Placebo	Phase 2
87	Not yet recruiting	Study to Evaluate a Single Intranasal Dose of STI-2099 (COVI-DROPS™) in Outpatient Adults With COVID-19 (US)	Covid19	• Biological: COVI-DROPS anti-SARS-CoV-2 spike neutralizing antibodies<br>• Drug: Placebo	Phase 2
88	Completed	SuPAR in Adult Patients With Covid-19	Covid19	• Diagnostic Test: Soluble Urokinase Plasminogen Activator Receptor	•
89	Recruiting	Study to Evaluate a Single Intranasal Dose of STI-2099 (COVI-DROPS™) in Outpatient Adults With COVID-19 (UK)	Covid19	• Biological: COVI-DROPS anti-SARS-CoV-2 spike neutralizing antibodies<br>• Drug: Placebo	Phase 2
90	Active, not recruiting	Study to Assess the Safety, Tolerability, and Pharmacokinetics of HFB30132A Against COVID-19 in Healthy Adults	Healthy	• Drug: HFB30132A *target* the HR1 domain<br>• Other: Placebo	Phase 1
91	Not yet recruiting	Efficiency and Security of NIVOLUMAB Therapy in Obese Individuals With COVID-19(COrona VIrus Disease) Infection	Obesity, COVID-19 Infection	• Drug: NIVOLUMAB (PD1-checkpoint inhibitor)<br>• Other: Routine standard of care	Phase 2
92	Recruiting	The Use of Tocilizumab in the Management of Patients Who Have Severe COVID-19 With	Covid19 Pneumonia	• Drug: Tocilizumab *anti-IL6 antibody*	Phase 4

		Suspected Pulmonary Hyperinflammation			
93	Completed<br>Has Results	Crizanlizumab for Treating COVID-19 Vasculopathy	COVID-19	• Drug: Crizanlizumab targets **selectin** to reduce the frequency of vasooclusive crises in patients with sickle cell disease<br>• Other: 0.9% saline	Phase 2
94	Recruiting	Efficacy of Reinforcing Standard Therapy in COVID-19 Patients With Repeated Transfusion of Convalescent Plasma	Covid19	• Other: Convalescent Plasma with antibody against SARS-CoV-2.<br>• Other: Standard treatment for COVID-19	Not Applicable
95	Recruiting	Ravulizumab and COVID-19	Covid19<br>Thrombotic Microangiopathies<br>Acute Kidney Injury	• Drug: Ravulizumab a long-acting, second-generation C5 inhibitor	Phase 3
96	Recruiting	Antibodies Production After Covid-19 Vaccination Among Patients With Medical History of Cancer and Anti-CD-20 Treatment	Covid19<br>Cancer<br>HIV Infections	• Diagnostic Test: Laboratory Samples	•
97	Recruiting	Outpatient Treatment With Anti-Coronavirus Immunoglobulin	COVID<br>SARS-CoV2 Infection<br>Covid19	• Biological: Hyperimmune immunoglobulin to SARS-CoV-2 (hIVIG)<br>• Other: Placebo	Phase 3
98	Not yet recruiting	Adrecizumab (HAM8101) to Improve Prognosis and Outcomes in COVID-19 Trial	COVID-19	• Biological: Adrecizumab (HAM 8101) Anti-Adrenomedullin Antibody<br>• Drug: Placebo	Phase 2<br>Phase 3
99	Active, not recruiting	Study of Mavrilimumab (KPL-301) in Participants Hospitalized With Severe Corona Virus Disease 2019 (COVID-19) Pneumonia and Hyper-inflammation	COVID	• Drug: mavrilimumab targeting GM-CSFR-α<br>• Other: Placebo	Phase 2<br>Phase 3
100	Withdrawn	CSL324 in COVID-19	Coronavirus Disease 2019 (COVID-19)	• Biological: CSL324 anti-G-CSFR antibody<br>• Drug: Placebo	Phase 2
101	Enrolling by invitation	COVID-19 and Multiple Sclerosis Disease Modifying Therapies	Covid19<br>Multiple Sclerosis		
102	Recruiting<br>New	A Safety and Tolerability Study of Sotrovimab (VIR-7831) Prophylaxis Against COVID-19 in Immunocompromised Individuals	SARS CoV 2 Infection	• Drug: Sotrovimab (targets an epitope in the RBD of the spike protein that is conserved between SARS-CoV and SARS-CoV-2)	Phase 2

103	Active, not recruiting	Randomized, Unicentric, Open, Controlled Clinical Trial, in Phase Iii, to Demonstrate the Effectiveness of Tocilizumab	COVID-19 Pneumonia	• Drug: Tociliziumab group *anti-IL6 antibody*<br>• Drug: Methylprednisolone group	Phase 3
104	Recruiting	Follow-up of Covid-19 Long Term Sequelae	COVID-19 Pneumonia<br><br>COVID-19 Respiratory Infection<br><br>COVID-19 Acute Bronchitis<br><br>(and 15 more...)	• Other: Not applicable (observational study)	
105	Not yet recruiting	Mavrilimumab in Severe COVID-19 Pneumonia and Hyper-inflammation (COMBAT-19)	Covid-19<br><br>Acute Respiratory Failure<br><br>ARDS, Human<br><br>(and 2 more...)	• Drug: Mavrilimumab targeting GM-CSFR-α<br>•<br>• Drug: Placebo	Phase 2
106	Completed	COVID-19 Long-Haulers Study	Coronavirus Disease 2019	• Drug: Placebos<br>• Drug: Leronlimab (700mg) bind CCR5	Phase 2
107	Withdrawn	Study to Evaluate STI-1499 (COVI-GUARD) in Patients With Moderate COVID-19	Covid-19	• Biological: COVI-GUARD anti spike protein<br>• Other: Standard of Care<br>• Drug: Placebo	Phase 1
108	Completed	Study to Evaluate the Efficacy and Safety of Leronlimab for Mild to Moderate COVID-19	Coronavirus Disease 2019	• Drug: Placebos<br>• Drug: Leronlimab (700mg) bind CCR5	Phase 2
109	Recruiting	ACTIV-5 / Big Effect Trial (BET-B) for the Treatment of COVID-19	COVID-19	• Biological: Lenzilumab a GM-CSF antagonist<br>• Other: Placebo<br>• Drug: Remdesivir	Phase 2
110	Active, not recruiting	Study to Evaluate the Efficacy and Safety of Leronlimab for Patients With Severe or Critical Coronavirus Disease 2019 (COVID-19)	Coronavirus Disease 2019	• Drug: Placebos<br>• Drug: Leronlimab (700mg) bind CCR5	Phase 2
111	Recruiting	Convalescent Plasma Therapy for Hospitalized Patients With COVID-19	COVID-19<br><br>Convalescent Plasma<br><br>SARS-CoV-2<br><br>(and 3 more...)	• Biological: Convalescent plasma<br>• Drug: Standard of care	Phase 2

112	Active, not recruiting	A Study to Assess if a Medicine Called Bamlanivimab is Safe and Effective in Reducing Hospitalization Due to COVID-19	Covid19	• Biological: Bamlanivimab that bind to different, but overlapping, epitopes in the spike protein RBD of SARS-CoV-2<br>• Other: Standard of Care	Phase 4
113	Not yet recruiting	Observational Study, Use of Canakinumab Administered Subcutaneously in the Treatment COVID-19 Pneumonia	COVID-19	• Drug: Canakinumab 150 MG/ML [Ilaris] binds to human IL-1β with high affinity	•
114	Completed	ACTIV-5 / Big Effect Trial (BET-A) for the Treatment of COVID-19	COVID-19	• Other: Placebo<br>• Drug: Remdesivir<br>• Biological: Risankizumab targeting IL- 23	Phase 2
115	Recruiting	mulTi-Arm Therapeutic Study in Pre-ICu Patients Admitted With Covid-19 - Repurposed Drugs (TACTIC-R)	COVID19	• Drug: Ravulizumab a long-acting, second-generation C5 inhibitor<br>•<br>• Drug: Baricitinib<br>• Other: Standard of care	Phase 4
116	Recruiting	Efficacy of COVID-19 Vaccination in Patientstreated With Anti-CD20 for Follicular Lymphoma or Mantle Cell Lymphoma	Follicular Lymphoma<br>Mantle Cell Lymphoma	• Biological: Determination of COVID-19 vacciantion efficacy	Not Applicable
117	Recruiting	PROSAIC-19 - Prospective Longitudinal Assessment in a COVID-19 Infected Cohort	COVID-19<br>SARS-CoV 2<br>ARDS, Human<br>Immune System Disorder	• Other: Blood tests sputum, nasal lavage and brushing	•
118	Completed<br>Has Results	Canakinumab in Covid-19 Cardiac Injury (The Three C Study)	COVID-19<br>SARS-CoV 2	• Drug: Canakinumab Injection 600mg<br>• Drug: Canakinumab Injection 300mg<br>• Drug: Placebos<br>binds to human IL-1β with high affinity	Phase 2
119	Completed	Management of Migraine Using Enerumab and Traditional Therapy at the Time of COVID-19	Migraine Disorders	• Drug: Erenumab targeting the CGRP pathway	•
120	Completed	Impact of the Covid-19 on RSV Epidemic	RSV Infection	• Other: Medical records analysis<br>• Other: Comparison of cohorts	•
121	Recruiting	Comparison of Tocilizumab Plus Dexamethasone vs. Dexamethasone for Patients With Covid-19	Coronavirus Infection	• Drug: Tocilizumab *anti-IL6 antibody*<br>• Drug: Dexamethasone	Phase 2

			SARS (Severe Acute Respiratory Syndrome)<br><br>Virus Diseases<br><br>(and 5 more...)		
122	Active, not recruiting	Cohort Multiple Randomized Controlled Trials Open-label of Immune Modulatory Drugs and Other Treatments in COVID-19 Patients - Sarilumab Trial - CORIMUNO-19 - SARI	Corona Virus Infection	• Drug: Sarilumab *targeted* against the alpha subunit of the IL-6 receptor complex	Phase 2<br>Phase 3
123	Terminated	Anti-il6 Treatment of Serious COVID-19 Disease With Threatening Respiratory Failure	Corona Virus Disease	• Drug: RoActemra iv<br>• Drug: RoActemra sc<br>• Drug: Kevzara sc<br>• Other: Standard medical care	Phase 2
124	Recruiting	Medical Herbs Inhibit Inflammation Directing T Cells to Kill the COVID-19 Virus (COVID)	Covid19 Virus Infection	• Dietary Supplement: Inflammation (I)<br>• Dietary Supplement: Inflammation (II)<br>• Dietary Supplement: Inflammation (III)<br>• Drug: Standard of care	Phase 1<br>Phase 2
125	Completed	Study of Multiple Candidate Agents for the Treatment of COVID-19 in Hospitalized Patients	COVID-19	• Drug: Standard of care<br>• Drug: Apremilast<br>• Drug: Apremilast placebo<br>• (and 4 more...)	Phase 3
126	Recruiting	National Project on Vaccines, COVID-19 and Frail Patients	COVID-19<br><br>Solid Tumor<br><br>Hematologic Diseases<br><br>(and 2 more...)	• Biological: COVID-19 vaccines	
127	Not yet recruiting	Understanding the Long-term Impact of COVID on Children and Families	SARS-CoV-2 Infection		
128	Recruiting	Severe Asthma Covid Vaccine Response Study	Coronavirus Antibody Levels	• Other: Not Interventional their treatments are their routine NHS care	
129	Active, not recruiting	CORIMUNO-19 - Tocilizumab Trial - TOCI (CORIMUNO-TOCI)	Corona Virus Infection	• Drug: Tocilizumab *anti-IL6 antibody*	Phase 2
130	Terminated	COVID Cohort Study	COVID-19<br><br>Acute Respiratory Failure	• Other: COVID-19+ observational	•

131	Active, not recruiting	Characterizing SARS-CoV-2-specific Immunity in Individuals Who Have Recovered From COVID-19	SARS-CoV-2<br><br>COVID-19	• Other: Sample collection	•
132	Recruiting	I-SPY COVID-19 TRIAL: An Adaptive Platform Trial for Critically Ill Patients	COVID-19	• Drug: Remdesivir<br>• Drug: Pulmozyme<br>• Drug: IC14 a monoclonal antibody against CD14<br>• (and 4 more...)	Phase 2
133	Recruiting	A Study on the Immune-response to COVID-19 Vaccination in Cancer Patients - the IOSI-COVID-19-001 Study	Covid19<br><br>Cancer	• Procedure: Blood sample	•
134	No longer available	SOLIRIS® (Eculizumab) Treatment of Participants With COVID-19	COVID-19<br><br>Pneumonia, Viral<br><br>Acute Lung Injury/Acute Respiratory Distress Syndrome (ARDS)	• Biological: eculizumab targets complement **protein C5**	•
135	Terminated	Efficacy and Safety Study of IV Ravulizumab in Patients With COVID-19 Severe Pneumonia	COVID-19 Severe Pneumonia<br><br>Acute Lung Injury<br><br>Acute Respiratory Distress Syndrome<br><br>Pneumonia, Viral	• Biological: Ravulizumab a long-acting, second-generation C5 inhibitor<br>• Other: Best Supportive Care	Phase 3
136	Recruiting	Randomised Evaluation of COVID-19 Therapy	Severe Acute Respiratory Syndrome	• Drug: Lopinavir-Ritonavir<br>• Drug: Corticosteroid<br>• Drug: Hydroxychloroquine<br>• (and 13 more...)	Phase 2<br>Phase 3
137	Recruiting	Quantification of Binding and Neutralizing Antibody Levels in COVID-19 Vaccinated Health Care Workers Over 1 Year	COVID-19 Vaccine<br><br>Corona Virus Infection<br><br>Vaccine Adverse Reaction<br><br>(and 3 more...)		
	Human Cytomegalovirus				
1	Completed	Efficacy Study of Human Cytomegalovirus (HCMV) Hyperimmune Globulin to Prevent Congenital HCMV Infection	Cytomegalovirus Infection	• Drug: HCMV-specific hyperimmune globulin (Cytotect®)<br>• Drug: Isotonic solution of sodium chloride (placebo)	Phase 2<br>Phase 3
2	Unknown †	The Link Between Human Cytomegalovirus (HCMV) and Hypertension	HCMV Infection<br><br>Hypertension	•	

3	Completed	Prospective, Randomized Study for Predicting Human Cytomegalovirus (hCMV) Infection Based on Baseline hCMV Specific T-cell Response in Kidney Transplant	CMV Infection<br><br>Kidney Transplantation	• Other: BIOMARKER- elispot test	Phase 4
4	Unknown †	Diaplacental Transfer of Anti-HCMV- and Anti-VZV-immunoglobulin G (IgG) - Antibodies at Premature and Mature Newborns	Materno-fetal Transfer of Anti-HCMV-IgG<br><br>Materno-fetal Transfer of Anti-VZV-IgG	• Other: Blood samples	•
5	Completed<br>Has Results	Efficacy and Safety Study of CSJ148 in Stem Cell Transplant Patients	HCMV	• Biological: CSJ148 *Target* Glycoprotein<br>• Drug: Placebo	Phase 2
6	Completed	Detection of Human Cytomegalovirus in the Saliva	Cytomegalovirus	•	
7	Completed<br>Has Results	Study to Evaluate Safety, Tolerability, and Immunogenicity of Candidate Human Cytomegalovirus Vaccine in Healthy Adults	Cytomegalovirus Infections	• Drug: VBI-1501A 0.5 µg<br>• Drug: VBI-1501A 1.0 µg<br>• Drug: VBI-1501A 2.0 µg<br>• (and 2 more...)<br><br>a vaccine candidate containing **a gB antigen**	Phase 1
8	Completed	Epidemiology of Human Cytomegalovirus Excretion in the Saliva of Children Attending Nursery in France	Healthy Volunteers	•	
9	Completed	The Epidemiologic Study of Human Cytomegalovirus （CMV） in Female Students of Xiamen University	Cytomegalovirus Infections	•	
10	Completed<br>Has Results	Safety and Immunogenicity of the Human Cytomegalovirus (CMV) Vaccine (V160) in Healthy Japanese Men (V160-003)	Cytomegalovirus Infections	• Biological: V160 (a live hCMV vaccine)<br>• Other: Placebo	Phase 1
11	Completed<br>Has Results	Safety, Tolerability, and Immunogenicity of the Human Cytomegalovirus Vaccine (V160) in Healthy Adults (V160-001)	Cytomegalovirus Infections	• Biological: V160 Low Dose IM<br>• Biological: V160 Medium Dose IM<br>• Biological: V160 High Dose IM<br>• (and 6 more...)	Phase 1
12	Completed	Artesunate in Preemptive Treatment of Human Cytomegalovirus (CMV) in Stem Cell Transplant Recipients	Cytomegalovirus Infections	• Drug: Artesunate	Phase 3
13	Active, not recruiting	MK-8228 (Letermovir) in the Prevention of Human Cytomegalovirus (CMV) Infection and Disease in Adult	Cytomegalovirus Infection	• Drug: Letermovir tablet<br>• Drug: Letermovir IV	Phase 3

		Japanese Kidney Transplant Recipients (MK-8228-042)	Cytomegalovirus Disease		
14	Active, not recruiting	Letermovir Versus Valganciclovir to Prevent Human Cytomegalovirus Disease in Kidney Transplant Recipients (MK-8228-002)	CMV Disease	• Drug: Letermovir<br>• Drug: Valganciclovir<br>• Drug: Acyclovir (ACV)<br>• (and 3 more...)	Phase 3
15	Unknown †	Epidemiology and Prevention of Congenital HCMV in Immune Mothers. Congenital HCMV Infection Lombardy	Congenital Cytomegalovirus Infection<br><br>Maternal Cytomegalovirus Infection	• Behavioral: Hygienic recommendations	Not Applicabl e
16	Complete d	Trial to Evaluate Safety and Immunogenicity of a Vaccine Against HCMV	Cytomegalovirus Infection	• Biological: Low dose HB-101<br>• Biological: Medium dose HB-101<br>• Biological: High dose HB-101<br>• Biological: Placebo	Phase 1
17	Unknown †	The Prevelance Rate of Human Cytomegalovirus (HCMV), Epstein-Barr Virus (EBV) and Human Herpes Virus (HHV-6) in a Saliva of the Patient With Periodantitis	Cytomegalovirus<br><br>Epstein-Barr Virus Infections<br><br>Herpesvirus Infection	•	
18	Complete d	Safety Study of Four Chimera Cytomegalovirus (CMV) Vaccines in Healthy Adult Males 30-50 Years of Age	Cytomegalovirus	• Biological: cmv vaccine	Phase 1
19	Complete d	Vaccine Therapy in Preventing Cytomegalovirus in Healthy Participants	Nonneoplastic Condition	• Biological: PADRE-CMV fusion peptide vaccine<br>• Biological: tetanus-CMV fusion peptide vaccine<br>• Drug: agatolimod sodium	Phase 1
20	Complete d	Cytomegalovirus Vaccine in Healthy Participants	Precancerous/Nonmalig nant Condition	• Biological: CMVpp65-A*0201 peptide vaccine	Phase 1
21	Not yet recruiting	Letermovir for CMV Prevention After Lung Transplantation	Lung Transplant<br><br>CMV	• Drug: Letermovir<br>• Drug: Valganciclovir	Phase 2
22	Complete d<br>Has Resul ts	Letermovir (MK-8228) Versus Placebo in the Prevention of Clinically-Significant Cytomegalovirus (CMV) Infection in Adult, CMV-Seropositive Allogeneic Hematopoietic Stem Cell Transplant Recipients (MK-8228-001)	Prevention of CMV Infection or Disease	• Drug: Letermovir<br>• Drug: Placebo	Phase 3
23	Suspende d	Cytomegalovirus (CMV) Transmission by Frozen Breast Milk in Preterms	Cytomegalovirus	•	

24	Completed	Effects of Hormone Therapy on the Immune Systems of Postmenopausal Women With Chronic Infections	Atherosclerosis<br><br>Chlamydia Infections<br><br>Cytomegalovirus Infections<br><br>(and 2 more...)	• Drug: Estrogen therapy	Phase 2
25	Completed	BCG and Plasma Amyloid in Non-Demented Adults	Alzheimer Disease, Late Onset	• Biological: Bacillus of Calmette and Guerin (BCG)	Phase 2
	Respiratory Syncytial Virus				
1	Active, not recruiting	A Study to Evaluate the Safety of MEDI8897 for the Prevention of Medically Attended Respiratory Syncytial Virus(RSV) Lower Respiratory Track Infection (LRTI) in High-risk Children	Respiratory Syncytial Virus Infections	• Drug: MEDI8897 targets the pre-F antigenic region<br>• Drug: Palivizumab targeting the **antigenic site II (also called site A) region of the RSV F protein**	Phase 2<br>Phase 3
2	Completed	Utilization and Compliance of Respiratory Syncytial Virus Monoclonal Antibody Therapy	Lower Respiratory Tract Infection<br><br>Respiratory Syncytial Virus Infections		
3	Active, not recruiting	A Study to Evaluate the Safety and Efficacy of MEDI8897 for the Prevention of Medically Attended RSV LRTI in Healthy Late Preterm and Term Infants	Respiratory Syncytial Virus Infections	• Drug: MEDI8897 targets the pre-F antigenic region<br>• Drug: Placebo	Phase 3
4	Completed<br>Has Results	Safety Study of a Monoclonal Antibody to Respiratory Syncytial Virus (RSV) in Children Hospitalized With RSV Infection	Respiratory Syncytial Virus Prophylaxis	• Biological: Motavizumab targeting RSV glycoprotein F<br>• Other: Placebo	Phase 1
5	Recruiting	Safety, Tolerability, and Pharmacokinetics of RSV Monoclonal Antibody RSM01 in Healthy Adults	RSV Infection	• Drug: RSM01<br>• Other: Placebo	Phase 1
6	Completed<br>Has Results	Effectiveness of Synagis (Palivizumab) Immunoprophylaxis in Preterm Infants With High Risk of Severe Respiratory Syncytial Virus (RSV) Infection	Respiratory Syncytial Virus Infection	• Biological: Synagis (palivizumab), monoclonal antibody for passive immunoprophylaxis against severe RSV disease administered according to usual clinical practice.	•
7	Completed<br>Has Results	A Study to Evaluate the Safety and Efficacy of MEDI8897 for the Prevention of Medically Attended RSV LRTI in Healthy Preterm Infants.	Respiratory Syncytial Virus Infections	• Drug: MEDI8897 targets the pre-F antigenic region<br>• Drug: Placebo	Phase 2
8	Completed<br>Has Results	Study to Evaluate the Efficacy and Safety of Suptavumab (REGN2222) for the Prevention of Medically Attended RSV (Respiratory	Respiratory Syncytial Virus Infections	• Drug: Suptavumab 30 mg/kg<br>• Drug: Placebo Matched to Suptavumab<br>• Drug: Suptavumab 30 mg/kg- 1 Dose	Phase 3

		Syncytial Virus) Infection in Preterm Infants		• Drug: Suptavumab 30 mg/kg - 2 Doses<br><br>respiratory syncytial virus fusion protein	
9	Completed<br>Has Results	Study of MEDI-524 (Motavizumab) for the Prophylaxis of Serious Respiratory Syncytial Virus (RSV) Disease in High-Risk Children	Respiratory Syncytial Virus Infections	• Biological: motavizumab (MEDI-524) targeting RSV glycoprotein F<br>• Biological: palivizumab targeting the **antigenic site II (also called site A) region of the RSV F protein**	Phase 3
10	Completed	Trial to Evaluate the Safety, Tolerability, and Pharmacokinetics of MEDI-524 in Healthy Adults	Respiratory Syncytial Virus Infections	• Biological: MEDI-524 (Numax-TM)	Phase 1
11	Completed	Open-Label, Dose-Escalation Study of 2 to 5 IM Doses of MEDI-524 at 3 mg/kg or 15 mg/kg; Children to be Followed for 90 Days After Their Last Dose of MEDI-524	Respiratory Syncytial Virus Infections	• Biological: Medi-524	Phase 1<br>Phase 2
12	Completed<br>Has Results	Study of Motavizumab (MEDI-524) and Palivizumab Administered Sequentially in the Same Respiratory Syncytial Virus (RSV) Season	Respiratory Syncytial Virus Infections<br><br>Chronic Lung Disease and <= 24 Months of Age or<br><br>Premature With Gestational Age <=35 Weeks and <=6 Months of Age	• Biological: Motavizumab RSV glycoprotein F, palivizumab targeting the **antigenic site II (also called site A) region of the RSV F protein**<br>• Biological: Palivizumab targeting the **antigenic site II (also called site A) region of the RSV F protein**, motavizumab targeting RSV glycoprotein F<br>• Biological: Motavizumab targeting RSV glycoprotein F	Phase 2
13	Completed<br>Has Results	A Phase 1 Study to Evaluate the Safety, Tolerability, and Pharmacokinetics of MEDI8897 in Healthy Adults	Respiratory Syncytial Virus Infections	• Drug: MEDI8897 Intravenous<br>• Drug: Placebo<br>• Drug: MEDI8897 Intramuscular<br><br>targets the pre-F antigenic region	Phase 1
14	Completed<br>Has Results	A Phase 1b/2a Randomized, Double-Blind, Placebo-controlled, Dose-escalation Study to Evaluate the Safety, Tolerability, and Pharmacokinetics of MEDI8897, a Monoclonal Antibody With an Extended Half-life Against Respiratory Syncytial Virus, in Healthy Preterm Infants	Respiratory Syncytial Virus	• Drug: Placebo<br>• Drug: MEDI8897 10 mg<br>• Drug: MEDI8897 25 mg<br>• Drug: MEDI8897 50 mg<br><br>targets the pre-F antigenic region	Phase 1
15	Completed	RSV001 - A New Vaccine to Prevent Severe Viral Chest Infections.	Respiratory Syncytial Virus Infections	• Biological: PanAd3-RSV given intra-nasally (high dose)<br>• Biological: MVA-RSV given by intra-muscular injection (high dose)	Phase 1

				• Biological: PanAd3-RSV given by intra-muscular injection (high dose)<br>• (and 3 more...)	
16	Completed	Impact of the Covid-19 on RSV Epidemic	RSV Infection	• Other: Medical records analysis<br>• Other: Comparison of cohorts	•
17	Completed	"Palivizumab Therapy for RSV-bronchiolitis"	Respiratory Syncytial Virus-bronchiolitis	• Drug: Palivizumab targeting the **antigenic site II (also called site A) region of the RSV F protein**<br>• Other: Placebo	Phase 3
18	Completed<br>Has Results	A Study to Assess the Safety and Effectiveness of Palivizumab Administered to Children at High Risk of Severe Respiratory Syncytial Virus (RSV) Infection in the Russian Federation and the Republic of Belarus	Respiratory Syncytial Virus (RSV)	• Drug: Palivizumab targeting the **antigenic site II (also called site A) region of the RSV F protein**	Phase 3
19	Completed	MEDI-557 Adult Dosing	Respiratory Syncytial Virus	• Drug: Placebo<br>• Drug: MEDI-557 targeting RSV glycoprotein F	Phase 1
20	Terminated<br>Has Results	A Study to Evaluate a Single Intramuscular Dose of Motavizumab to Treat Children With Respiratory Syncytial Virus (RSV) Illness	Participants Less Than 12 Months of Age With RSV Illness	• Biological: Motavizumab targeting RSV glycoprotein F Other: Placebo	Phase 2
21	Completed<br>Has Results	A Study to Evaluate a Single Intravenous Dose of Motavizumab for the Treatment of Children Hospitalized With Respiratory Syncytial Virus (RSV) Illness	RSV Illness in ≤12 Months of Participants	• Biological: Motavizumab targeting RSV glycoprotein F<br>• Other: Placebo	Not Applicable
22	Recruiting	Evaluate the Safety and Efficacy of Nirsevimab in Healthy Preterm and Term Infants in China	Lower Respiratory Tract Infection	• Drug: Nirsevimab target F glycoprotein of RSV<br>• Drug: Placebo	Phase 3
23	Completed<br>Has Results	MEDI-524 (Motavizumab) for the Prevention of Respiratory Sycytial Virus (RSV) Disease Among Native American Indian Infants in the Southwestern United States	Healthy	• Biological: Motavizumab targeting RSV glycoprotein F<br>• Other: Placebo	Phase 3
24	Completed<br>Has Results	A Study to Evaluate MEDI-524 In Children With Hemodynamically Significant Congenital Heart Disease	Congenital Heart Disease	• Biological: Motavizumab targeting RSV glycoprotein F<br>• Biological: Palivizumab targeting the **antigenic site II (also called site A) region of the RSV F protein**	Phase 2

25	Completed	A Phase I, Randomized, Double-Blind, Single-Dose, Dose Escalation Study to Evaluate the Safety, Tolerability, and Pharmacokinetics of MEDI-557	Healthy	• Biological: MEDI-557 targeting RSV glycoprotein F	Phase 1
26	Completed<br>Has Results	A Study to Evaluate the Safety, Tolerability, and Immunogenicity of Motavizumab (MEDI-524) After Dosing for a Second Season in Children	Motavizumab Administration for a Second Season for RSV Prophylaxis	• Biological: motavizumab (MEDI-524) targeting RSV glycoprotein F<br>• Biological: palivizumab 15 mg/kg targeting the **antigenic site II (also called site A) region of the RSV F protein**	Phase 1<br>Phase 2
27	Completed	A Phase II Randomized, Double-Blind, Two-Period Cross-Over Study to Evaluate the Pharmacokinetics, Safety and Tolerability of a Liquid Formulation of Palizvizumab (MEDI-493, Synagis)	-Unhealthy Children With a History of Prematurity	• Drug: MEDI-493 targeting the **antigenic site II (also called site A) region of the RSV F protein**	Phase 2
28	Completed<br>Has Results	Compliance to Synagis (Palivizumab) Under Daily Pediatrician´s Conditions in Premature Infants 33 - 35 wGA	Premature Infants		
	**(MERS-CoV) Middle East respiratory syndrome–related coronavirus**, a member of the genus Betacoronavirus ((+)ssRNA viruses, Group IV)				
1	Completed	A Safety, Tolerability, Pharmacokinetics and Immunogenicity Trial of Co-administered MERS-CoV Antibodies REGN3048 and REGN3051	Corona Virus Infection	• Other: Placebo<br>• Biological: REGN3048<br>• Biological: REGN3051<br><br>*target* the MERS coronavirus (MERS CoV) spike protein	Phase 1
